# Inaugurating High‐Throughput Profiling of Extracellular Vesicles for Earlier Ovarian Cancer Detection

**DOI:** 10.1002/advs.202301930

**Published:** 2023-07-23

**Authors:** Ala Jo, Allen Green, Jamie E. Medina, Sonia Iyer, Anders W. Ohman, Eric T. McCarthy, Ferenc Reinhardt, Thomas Gerton, Daniel Demehin, Ranjan Mishra, David L. Kolin, Hui Zheng, Jinwoo Cheon, Christopher P. Crum, Robert A. Weinberg, Bo R. Rueda, Cesar M. Castro, Daniela M. Dinulescu, Hakho Lee

**Affiliations:** ^1^ Center for Systems Biology Massachusetts General Hospital Harvard Medical School Boston MA 02114 USA; ^2^ Department of Radiology Massachusetts General Hospital Harvard Medical School Boston MA 02114 USA; ^3^ Center for Nanomedicine Institute for Basic Science Seoul 03722 Republic of Korea; ^4^ Division of Women's and Perinatal Pathology Department of Pathology Brigham and Women's Hospital Harvard Medical School Boston MA 02115 USA; ^5^ Whitehead Institute Massachusetts Institute of Technology Cambridge MA 02142 USA; ^6^ Biostatistics Center Massachusetts General Hospital Boston MA 02114 USA; ^7^ Division of Gynecologic Oncology Department of Obstetrics and Gynecology Massachusetts General Hospital Boston MA 02114 USA; ^8^ Cancer Center Massachusetts General Hospital Harvard Medical School Boston MA 02114 USA

**Keywords:** diagnostics, extracellular vesicles, ovarian cancer

## Abstract

Detecting early cancer through liquid biopsy is challenging due to the lack of specific biomarkers for early lesions and potentially low levels of these markers. The current study systematically develops an extracellular‐vesicle (EV)‐based test for early detection, specifically focusing on high‐grade serous ovarian carcinoma (HGSOC). The marker selection is based on emerging insights into HGSOC pathogenesis, notably that it arises from precursor lesions within the fallopian tube. This work thus establishes murine fallopian tube (mFT) cells with oncogenic mutations and performs proteomic analyses on mFT‐derived EVs. The identified markers are then evaluated with an orthotopic HGSOC animal model. In serially‐drawn blood of tumor‐bearing mice, mFT‐EV markers increase with tumor initiation, supporting their potential use in early cancer detection. A pilot clinical study (*n* = 51) further narrows EV markers to five candidates, EpCAM, CD24, VCAN, HE4, and TNC. The combined expression of these markers distinguishes HGSOC from non‐cancer with 89% sensitivity and 93% specificity. The same markers are also effective in classifying three groups (non‐cancer, early‐stage HGSOC, and late‐stage HGSOC). The developed approach, for the first time inaugurated in fallopian tube‐derived EVs, could be a minimally invasive tool to monitor women at high risk of ovarian cancer for timely intervention.

## Graphical abstract

This study utilizes extracellular vesicles (EVs) to detect high‐grade serous ovarian carcinoma (HGSOC) at an early stage. It capitalizes on insights into HGSOC's development from precursor lesions in the fallopian tube (FT). EV markers specific to FT tumors are identified and validated through a pilot clinical study. The findings highlight the potential of EV‐based tests for early cancer detection.

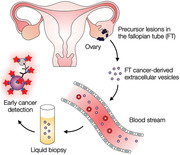

## 1 Introduction

Analyzing circulating biomarkers (liquid biopsy) is a compelling strategy in cancer management, empowering clinicians to detect and monitor diseases through minimally invasive and repeatable testing. Its transformative potential has been demonstrated, particularly in caring for patients with advanced diseases. Expanding liquid biopsy to early cancer, however, faces technical challenges. Information is limited on biomarkers specific to early lesions, and levels of these markers are presumably low in circulation. Such diagnostics challenges are evident with ovarian cancer (OvCa), the most lethal gynecological disease.^[^ ^1^ ^]^ Conventional blood testing (e.g., CA125) and imaging have failed to demonstrate survival advantages in a large, multi‐year screening trial,^[^ ^2^ ^]^ mainly because the most common OvCa subtype, high‐grade serous ovarian carcinoma (HGSOC), often presents after spreading beyond the primary site of origin. This fact underscores the need for improved detection methods and informed marker selection based on carcinogenesis and tumor evolution.^[^ ^2^, ^3^, ^4^ ^]^

Amassing biological and clinical data support that the bulk of HGSOC arises from precursor lesions within the distal fallopian tube (FT).^[^ ^5^, ^6^, ^7^, ^8^, ^9^, ^10^, ^11^ ^]^ Patients with advanced HGSOC frequently harbor serous tubal intraepithelial carcinoma (STIC) lesions with identical *TP53* mutations found in tumors. Statistical studies indicate that nearly 60% of epithelial HGSOCs are tubal in origin.^[^ ^5^, ^6^, ^7^, ^12^, ^13^ ^]^ This mechanistic insight raises the prospect of early HGSOC diagnostics by interrogating molecular markers derived from FT precursor lesions.

An appealing analytical target is extracellular vesicles (EVs) secreted by cells.^[^ ^14^, ^15^ ^]^ EVs reflect the molecular cargo of tumor cells and circulate in easily accessible bodily fluids.^[^ ^16^, ^17^, ^18^, ^19^, ^20^, ^21^, ^22^, ^23^ ^]^ Analyzing EVs can thus represent a real‐time, minimally invasive modality to detect and monitor tumor molecular status, including precursor lesions such as STICs.^[^ ^21^, ^24^, ^25^ ^]^ Already, tumor‐associated EVs have been demonstrated to be effective surrogate OvCa biomarkers for tumor detection and treatment monitoring.^[^ ^26^, ^27^ ^]^ Most studies, however, mainly analyzed late‐stage diseases, skewing EV markers toward advanced clinical presentations.^[^ ^25^, ^27^, ^28^, ^29^ ^]^ Conceivably, early HGSOC lesions may have molecular profiles distinct from those of late‐stage diseases or cell cultures – identifying and validating such EV signatures is crucial to improving early diagnosis.^[^ ^30^ ^]^

**Scheme 1:**
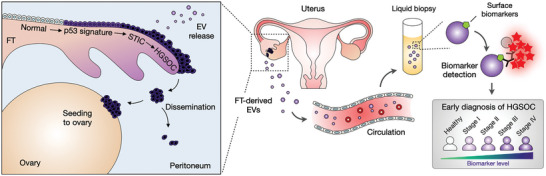
Study design. High‐grade serous ovarian cancer (HGSOC) is considered to arise from precursor lesions within the fallopian tube (FT). Circulating EVs from FT precursor lesions thus can serve as early HGSOC biomarkers. In this study, we identified EV markers specific to FT carcinoma and detected them in the blood samples of HGSOC patients. Analyzing surface markers on FT‐derived EVs allowed for differentiating early‐ (stage I & II) and late‐stage (stage III & IV) HGSOC patients.

Here, we report on our systematic approach to developing an EV‐based blood test for early‐stage (or low‐volume) HGSOC detection. We specifically reasoned that EVs from precursor lesions could be identified in blood to serve as circulating biomarkers for pre‐invasive/early‐stage HGSOC (**Scheme** **1**). To test this hypothesis, we took a two‐pronged approach – we developed a high‐throughput EV assay, termed SAViA (Signal Amplifying Vesicles in Array), and inaugurated the first known analyses of EVs derived from fallopian tubes. Combining EV physisorption and tyramide‐assisted signal enhancement, the SAViA assay increased analytical sensitivity by more than 1000‐fold over conventional immunoassays, allowing us to detect a low number of EVs (≈600 vesicles) in a convenient microwell‐plate format (386 wells). We next identified HGSOC‐specific EV markers by establishing FT tumor cells and analyzing their EVs via proteomics. These markers were then serially monitored in orthotopic HGSOC mouse models that mimicked tumor initiation, progression, and metastasis. The SAViA assay revealed that EVs expressing HGSOC markers increased with tumor initiation, supporting EVs’ potential use in early cancer detection. In the ensuing pilot study with clinical samples (*n* = 51), we further refined the HGSOC‐EV signature (CD24, EpCAM, HE4, TNC, VCAN) to achieve a diagnostic sensitivity of 0.89 and specificity of 0.93. The same five markers also effectively classified three clinically distinct groups: non‐cancer (*n* = 14), early‐stage HGSOC (stage I, II; *n* = 17), and late‐stage HGSOC (stage III, IV; *n* = 20). In particular, they differentiated early‐stage HGSOC from the rest with a specificity of 0.91 ( = 31/34).

## 2 Results

### 2.1 Optimizing the SAViA Assay

**Figure 1:**
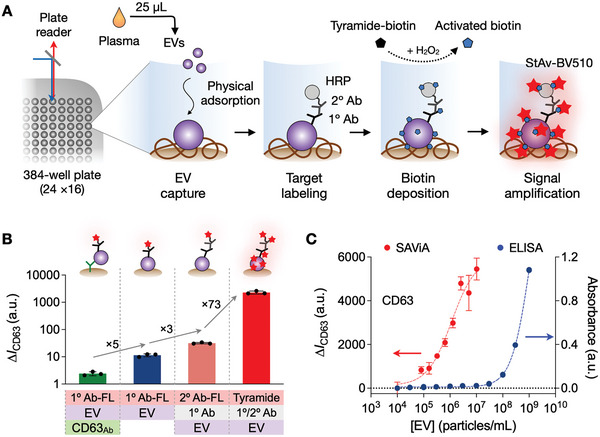
Sensitive, high‐throughput EV assay. A) SAViA (signal amplifying vesicles in array) scheme. EVs are captured on a multiwell plate via physical adsorption. Target EV protein is labeled with a primary antibody (1° Ab) which is further labeled with a secondary antibody (2° Ab) conjugated with horseradish peroxidase (HRP). With tyramide‐biotin and hydrogen peroxide (H_2_O_2_) added, the HRP catalyzes the dense deposition of biotin. Finally, the analytical signal is detected by adding fluorescent streptavidin (StAv‐BV510). B) Different EV‐assay formats were compared for the analytical signal. Physisorptive EV immobilization produced a higher signal (fivefold) than Ab‐based EV capture (left). Among the physisorptive EV assays, applying the tyramide amplification generated the highest signal. The overall signal increase was about 10^3^‐fold. Data are displayed as mean ± s.d. (*n* = 3). FL, fluorescent. C) The SAViA assay displayed superior sensitivity compared to conventional ELISA. Based on CD63 titration curves, the estimated detection limits were 2.4 × 10^4^ EV mL^−1^ for SAViA and 3.0 × 10^8^ EV mL^−1^ for ELISA. a.u., arbitrary units. Data are displayed as mean ± s.d. from technical triplicates.

We designed SAViA to enable EV assays with superior sensitivity and throughput, which is critical to detecting multiple proteins in low‐abundant EVs from early cancer lesions. **Figure** **1**A summarizes the assay scheme. We directly immobilized EVs on polystyrene microplate surfaces through passive adsorption (see Experimental Section for details). The binding was primarily mediated by hydrophobic interactions, and it allowed for unbiased EV capture regardless of the expression of tetraspanins (CD63, CD9, CD81), the canonical EV markers. We then applied tyramide signal amplification to boost the detection sensitivity. Target EV proteins were labeled with primary antibodies, which were further labeled with horseradish peroxidase (HRP) through secondary antibodies. When tyramide‐biotin and H_2_O_2_ were added, HRP‐labeled EVs catalyzed the production of reactive tyramide radicals, triggering the dense deposition of biotin molecules on nearby tyrosine residues.^[^ ^31^ ^]^ Finally, fluorescent streptavidin was coupled to the biotin deposit for signal generation.

The SAViA assay produced the highest analytical signal among the different assay configurations tested (Figure 1B). As a model system, we probed tumor‐cell derived EVs for CD63 expression, measuring the net fluorescence intensity, Δ*I*_CD63_ = *I*_CD63_ – *I*_IgG_, wherein *I*_CD63_ and *I*_IgG_ were, respectively, the signals from samples labeled with a CD63 antibody and an isotype IgG antibody. Comparing EV‐immobilization results, we observed a fivefold signal increase when switching from the antibody‐based EV capture to physisorption. The signal further improved (threefold) when a fluorescent secondary antibody was used for labeling, and the subsequent amplification led to an additional 73‐fold signal boost. With this large signal gain (>1000‐fold overall), the SAViA assay achieved superb analytical sensitivity. Varying the input EV loading, we measured Δ*I*_CD63_ (Figure 1C). SAViA's limit of detection (2.4 × 10^4^ EV mL^−1^; 6.0 × 10^2^ EVs in 25 µL) was notably lower than that of conventional sandwich‐type ELISA (3.0 × 10^8^ EV mL^−1^; 1.5 × 10^7^ EVs in 50 µL). Such high sensitivity facilitated marker detection within a small volume (25 µL) of plasma, enabling us to adopt a convenient 384‐well plate format for high throughput.

### 2.2 Generating Murine Fallopian Tube‐Derived Tumor Cell Lines

**Figure 2:**
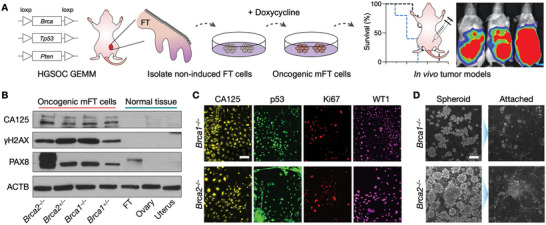
Generation and characterization of mFT cell lines. (A) FT cells were isolated from genetically engineered mice harboring mutations in *Brca1* or *Brca2*, as well as *Tp53* and *Pten*. Isolated cells were rendered oncogenic through the doxycycline treatment. Tumor animal models were generated by implanting the transformed cells into mice. GEMM, genetically engineered mouse model. B) Oncogenic mFT cells expressed FT‐specific protein (PAX8) and HGSOC markers (CA125, γH2AX). Normal tissue samples (uterus, ovary, FT) lacked HGSOC markers, while PAX8 was positive only with FT tissue. C) Immunofluorescence microscopy confirmed that the oncogenic mFT cells (mFT3635 and mFT3666) expressed key HGSOC markers (CA125, p53, Ki67, WT1) at the cellular level. Scale bar, 50 µm. D) Under in vitro ultra‐low adherence culture conditions, oncogenic mFT cells (mFT3635 and mFT3666) formed tumor spheroids (left). When transferred to adhesion plates, tumor spheroids adhered to the surface and spread, demonstrating their capacity to engraft. Scale bar, 100 µm.

We next established murine fallopian tube (mFT) tumor cell lines from genetically engineered mouse models (GEMMs) of HGSOC^[^ ^32^ ^]^ (**Figure** **2**A). The GEMMs contained a *Pax8‐Cre* transgene and different combinations of *Brca* (*Brca1* or *Brca2*), *Tp53*, and *Pten* floxed genes. Under these constructs, Cre‐recombinase expression can be driven by the *Pax8* promoter; PAX8 is a transcription factor specific to Müllerian‐derived epithelia (e.g., FT) but not the ovaries.^[^ ^33^ ^]^ We isolated non‐induced mFT cells from GEMM cohorts and treated the cells with doxycycline, inactivating target genes via Cre‐mediated recombination. These processes produced oncogenic mFT cell lines with different genotypes: mFT3707 (*Brca1^+/−^*, *Tp53^mut^*, *Pten^−/−^*), mFT3635 (*Brca1^−/−^*, *Tp53^mut^*, *Pten^−/−^*), mFT3665 (*Brca2^+/−^*, *Tp53^mut^*, *Pten^−/−^*), and mFT3666 (*Brca2^−/−^*, *Tp53^mut^*, *Pten^−/−^*). We further transfected the cell lines with mCherry/luciferase plasmid and sorted them according to mCherry expression (see Experimental Section for details).

The transformed mFT cell lines had their intended *Brca*, *Pten*, and *Tp53* genotypes verified by targeted polymerase chain reactions (Figure S1, Supporting Information). At the protein level, the mFT cell lines maintained the expression of the FT epithelial marker (PAX8), but they acquired *de novo* expression of HGSOC markers (CA125, γ‐H2AX) that were absent in normal uterine, ovarian, and FT tissues (Figure 2B).^[^ ^34^, ^35^ ^]^ Immunofluorescence imaging further confirmed that the mFT cell lines expressed key HGSOC markers (CA125, TP53, Ki67, WT1; Figure 2C and Figure S2A, Supporting Information).^[^ ^35^, ^36^, ^37^ ^]^ When cultured in vitro on ultra‐low adhesion plates, the transformed mFT cells formed spheroids. When these spheroids were re‐introduced to adherent conditions, they bound to a surface and formed a monolayer, demonstrating their potential to engraft and grow into tumors (Figure 2D and Figure S2B, Supporting Information).

### 2.3 Defining mFT‐EV Marker Candidates

**Figure 3:**
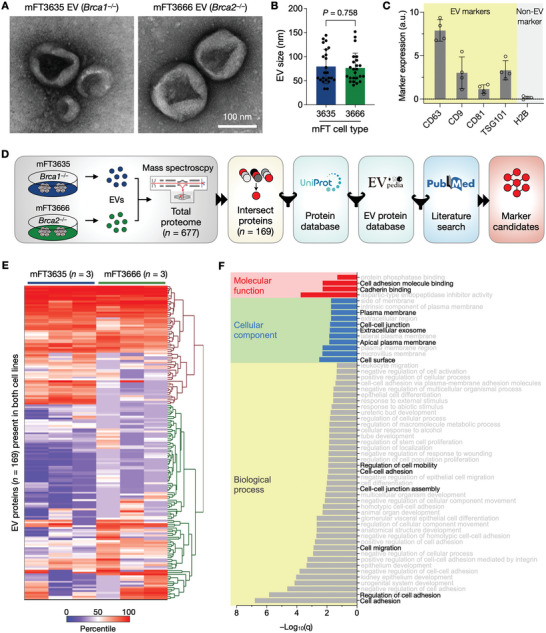
mFT‐EV marker selection. A) EVs from oncogenic mFT cells were imaged via transmission electron microscopy (TEM). B) Size distribution of EVs in TEM images. No significant difference (*P* = 0.758, unpaired two‐sided *t‐*test) was observed. Each dot represents a single EV. Error bars, s.d. C) Bulk EV analysis confirmed that mFT EVs were enriched with canonical EV markers (i.e., CD63, CD9, CD81, TSG101) and devoid of a non‐EV marker (histone H2B), a.u., arbitrary units. Data are displayed as mean ± s.d. (*n* = 4). D) Marker selection algorithm. EVs from mFT3635 and mFT3666 cells were processed for proteomic analysis. Detected proteins were filtered for their location in the cell membrane (Uniprot) and presence in EVs (EVpedia), and the outcomes were further curated through a literature search (PubMed). E) Heatmap of proteins (*n* = 169) found in EVs from mFT3635 and mFT3666 cell lines. The data‐driven approach selected nine candidate markers (PODXL, JUP, TNC, VCAN, CD24, EpCAM, HE4, FOLR1, and CA125). F) Gene ontology (GO) analysis showed that the selected markers were strongly associated with cellular adhesion. GO analysis was performed with STRING v11.5.

To determine protein candidates for mFT tumors, we analyzed EVs derived from oncogenic mFT cell lines with two distinct genotypes, mFT3635 (*Brca1^−/−^*) and mFT3666 (*Brca2^−/−^*). Cells were cultured in identical conditions, and vesicles in the culture media were collected via size exclusion chromatography (see Experimental Section). We observed no significant difference in physical profiles between these two sample types. Vesicles displayed a similar morphology under electron microscopy (**Figure** **3**A and Figure S3, Supporting Information), with a size range of 50–150 nm (Figure 3B). The samples were positive for canonical EV markers (i.e., CD63, CD9, CD81, TSG101) and devoid of a non‐EV marker (i.e., histone H2B), confirming EV's presence (Figure 3C).

We next carried out comprehensive EV proteomic analyses for marker discovery. EVs from mFT3635 and mFT3666 cells were subjected to liquid chromatography‐mass spectrometry, and the results were processed through a bioinformatic pipeline (Figure 3D). The mass spectrometry identified 677 proteins from six replicates (see Supplementary Data): 631 proteins from mFT3635‐EVs and 215 proteins from mFT3666‐EVs (Figure S4, Supporting Information). We selected 169 proteins found in both EV types (Figure 3E), effectively identifying markers relevant to both *Brca1/2* mutations. We further narrowed the list by referencing public databases: i) cell proteome data (UniProt) for membrane‐associated proteins and ii) an established EV proteome database (EVpedia) for markers present in EVs. We finally augmented the list with additional protein markers from the literature: human epididymis protein 4 (HE4), cancer antigen 125 (CA125), CD24, and epithelial cell adhesion molecule (EpCAM). Both HE4 and CA125 are known serum markers for OvCa diagnosis,^[^ ^38^, ^39^ ^]^ and CD24 and EpCAM are shown to be overexpressed in OvCa EVs.^[^ ^27^, ^29^, ^40^ ^]^

The selection algorithm produced the final nine candidate markers: podocalyxin (PODXL), junction plakoglobin (JUP), tenascin‐C (TNC), versican (VCAN), folate receptor 1 (FOLR1), CD24, EpCAM, and CA125. Gene ontology analysis confirmed the enrichment of these nine markers in the plasma membrane and EVs (Figure 3F). The dominant function was cellular adhesion (PODXL, JUP, TNC, VCAN, CD24, EpCAM),^[^ ^41^, ^42^, ^43^, ^44^, ^45^, ^46^ ^]^ followed by immune responses within cells (HE4, CA125)^[^ ^39^, ^47^ ^]^ and DNA repair (FOLR1).^[^ ^48^ ^]^ We validated the expression of the candidate markers in mFT parental cells (Figure S5A, Supporting Information). All markers stained positive in oncogenic mFT cell lines. Interestingly, these markers were also positive in cells collected from HGSOC mice ascites (Figure S5B, Supporting Information), which suggests they may be expressed throughout disease progression.

### 2.4 Serial Profiling of Plasma EVs from mFT‐Tumor Mice

**Figure 4:**
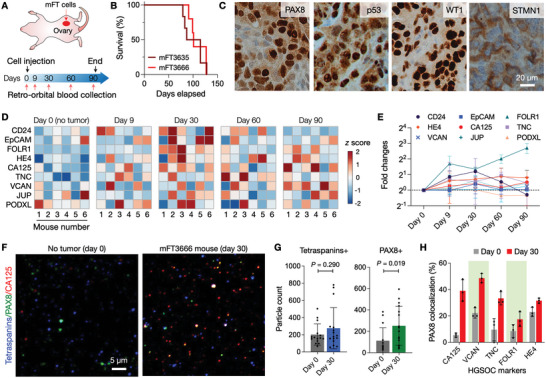
Serial EV profiling with an HGSOC animal model. A) Study design. Oncogenic mFT cells were implanted into ovary fat mass in mice. Blood samples were collected from the animals before engraftment and up to 3 months thereafter. For each sample, EVs were collected and profiled by SAViA for nine HGSOC candidate markers. B) Survival analysis of mFT3635 (*Brca1* ^−/−^) and mFT3666 (*Brca2* ^−/−^) implanted animals. No significant difference (*P* = 0.989; log‐rank test) in survival was observed. The median survival was 107 (mFT3635, *n* = 6) and 114 days (mFT3666, *n* = 6). C) Immunohistochemical staining confirmed the expression of the FT epithelial (PAX8) and tumor markers (p53, WT1, STMN1) in a late‐stage (day 90) tumor. D) Longitudinal EV profiling in tumor‐bearing animals (*n* = 6). Nine HGSOC markers were measured in plasma EVs. The heatmap shows the z‐score of each marker. E) The expression of HGSOC markers increased after tumor initiation (day 9) and peaked 30 days after the mFT cell implant. Each data point is the average of fold changes from six animals. Data are displayed as mean ± s.d. (*n* = 6). F) Single EVs were imaged in plasma samples collected before the mFT cell engraftment (day 0) and during disease progression (day 30). EVs were stained for tetraspanins (CD63, CD9), PAX8 (FT epithelial marker), and CA125 marker (see Figure S8, Supporting Information for other markers). G) Tetraspanin‐positive EVs were present in both samples (day 0 and day 30) with no significant difference in numbers (*P* = 0.290; non‐paired, two‐sided *t*‐test). PAX8‐positive EVs, however, significantly increased in the tumor sample (*P* = 0.019; non‐paired, two‐sided *t*‐test). Data are displayed as mean ± s.d. (*n* = 15 field of views). H) EV imaging revealed that more EVs were both PAX8 and HGSOC‐marker positive in tumor samples. Data are displayed as mean ± s.d. (*n* = 3). The *P*‐values (non‐paired, two‐sided *t*‐test) are 0.002 (CA125), 0.0009 (VCAN), 0.014 (TNC), 0.105 (FOLR1), and 0.018 (HE4).

To test the potential of EV markers in early HGSOC detection, we monitored them in a longitudinal mouse study (**Figure** **4**A). To replicate mFT tumor initiation and progression in the ovary, we implanted oncogenic mFT cells into the ovarian fat pad/bursa of NSG mice. Both mFT cell types, mFT3635 (*Brca1^−/−^*) and mFT3666 (*Brca2^−/−^*), developed into a tumor with no significant difference (*P* = 0.817; log‐rank test) in survival rates (Figure 4B). When these cell lines were implanted intraperitoneally (IP) into NOD SCID mice, the tumor spread throughout the peritoneal cavity and caused extensive ascites (Figure S6, Supporting Information), recapitulating in situ and late‐stage diseases. Immunohistochemical staining revealed that the late‐stage tumor (IP‐engrafted) was positive for PAX8 (FT epithelial marker),^[^ ^33^ ^]^ TP53 (HGSOC marker),^[^ ^36^ ^]^ WT1 (HGSOC marker),^[^ ^37^ ^]^ and STMN1 (tumor progression),^[^ ^49^ ^]^ confirming the tumor's mFT origin and metastatic potential (Figure 4C).

For longitudinal EV profiling, we collected serial blood samples from the host animals (*n* = 6) starting before engraftment and continuing up to 3 months thereafter. The SAViA assay was then applied to detect mFT markers in plasma EVs (Figure 4D; see Experimental Section for details). The overall expression of mFT markers elevated in all animals after tumor initiation (Day 9) and peaked 30 days after the mFT cell implant (Figure 4E). Moreover, we observed no significant differences in the marker expression between mFT3635‐ and mFT3666‐implanted animal cohorts (Figure S7, Supporting Information). Together, the results supported EVs’ potential for early HGSOC detection.

We further validated the presence of mFT‐derived EVs in blood by performing single EV imaging of plasma samples collected before mFT‐cell implantation (Day 0) and during tumor growth (Day 30). Samples were stained for tetraspanins (CD63, CD9; EV identification), an FT epithelial marker (PAX8), and HGSOC markers (Figure 4F). No significant changes were observed in tetraspanin‐positive EV numbers between no‐tumor (Day 0) and tumor (Day 30) samples, whereas PAX8‐positive EV numbers significantly increased in the tumor samples (Figure 4G). Notably, more EVs were PAX8 and HGSOC‐marker positive in tumor samples (Figure 4H and Figure S8, Supporting Information), validating the presence of FT‐derived tumor EVs in circulation.

### 2.5 Profiling Plasma EVs from Clinical Samples

**Figure 5:**
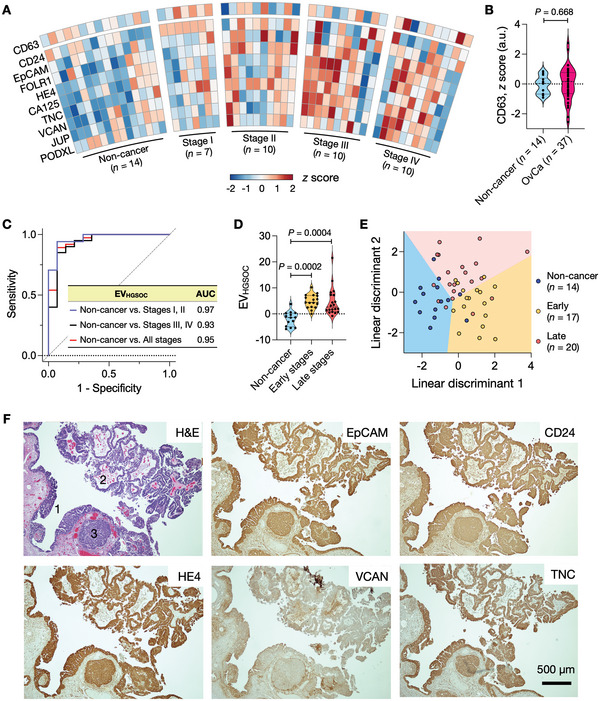
Profiling of plasma EVs from HGSOC patients. A) EVs from clinical plasma samples were profiled for HGSOC markers and CD63 (*n* = 14, non‐cancer individuals; *n* = 37, HGSOC patients). The expression of each marker was normalized (z‐score) and displayed in a heatmap. B) CD63 measurements confirmed the presence of EVs both in non‐cancer and HGSOC plasma samples. No significant difference was observed in CD63 expression between the two cohorts (*P* = 0.688; non‐paired, two‐sided *t*‐test). C) Five markers (EpCAM, CD24, HE4, VCAN, TNC) were chosen from a regression analysis, and their expressions were combined to define the EV_HGSOC_ score. In the receiver operating characteristic (ROC) analysis, the EV_HGSOC_ score achieved high accuracies in differentiating HGSOC patients from non‐cancer individuals. AUC, an area under the curve. D) EV_HGSOC_ scores were higher in early and late‐stage HGSOC patients than in non‐cancer individuals but were similar among HGSOC cohorts (Tukey's multiple comparisons test). Early, stages I & II; Late, stages III & IV. E) Linear discriminant analysis (LDA) model of top‐five markers (EpCAM, CD24, HE4, VCAN, TNC) differentiated three groups: non‐cancer individuals, early‐stage patients, and late‐stage patients. The overall classification accuracy was 74.5%. F) Tumor tissue of HGSOC patients was stained positive for HGSOC markers (EpCAM, CD24, HE4, VCAN, TNC). In the H&E micrograph, annotated are STIC lesions (1) and HGSOC (2 and 3). See Figure S12, Supporting Information, for other HGSOC markers.

We next conducted a pilot study to evaluate FT‐derived EV markers in human clinical samples (**Table** **1**). Plasma samples were obtained from HGSOC patients (*n* = 37) at different stages (*n* =7, Stage I; *n* = 10, Stage II; *n* = 10, Stage III; *n* = 10, Stage IV) and from non‐cancer female donors (*n* = 14). We isolated EVs and subjected them to the SAViA assay, analyzing the expression of the nine candidate markers along with CD63 (**Figure** **5**A and Figure S9, Supporting Information). All samples showed positive CD63 values (above the control IgG level) to validate EV presence. Yet no significant difference (*P* = 0.668; unpaired two‐sided *t*‐test) was observed between the non‐cancer and HGSOC cohorts (Figure 5B).

**Table 1:** Clinical information of patients

Case	Non‐cancer	HGSOC
	14	37
**Age**		
Median	52	62
Range	23–72	42–82
**Sex**		
Female	14 (100%)	37 (100%)
**Stage**		
I	‐	7 (19%)
II	‐	10 (27%)
III	‐	10 (27%)
IV	‐	10 (27%)
**BRCA status**		
Not tested	‐	20 (54%)
BRCA1 mutant	‐	4 (11%)
BRCA2 mutant	‐	3 (8%)
Negative	‐	10 (27%)
**Serum marker (median)**		
CA125 (U mL^−1^)	‐	659.2 (11–12 561)^a)^

^a)^ Values indicate ranges.

We first focused on overall HGSOC diagnostics through circulating EV analyses. Informed by our EV profiling results, we narrowed down the nine HGSOC candidate markers to top‐five proteins (i.e., EpCAM, CD24, HE4, VCAN, TNC) via lasso (least absolute shrinkage and selection operator) analyses. We then defined an EV_HGSOC_ score by combining the expression of these five markers through logistic regression (see Experimental Section for detail). The EV_HGSOC_ score achieved high accuracy for HGSOC detection (sensitivity, 0.89; specificity, 0.93) with an area under the curve (AUC) of 0.95 (Figure 5C). The EV_HGSOC_ score also outperformed single markers and even the nine‐marker combination (see Table S1 and Figure S10, Supporting Information for comparison). We further compared EV_HGSOC_ scores among three groups: non‐cancer individuals (*n* = 14), early‐stage (I & II) HGSOC (*n* = 17), and late‐stage (III & IV) HGSOC (*n* = 20). The average EV_HGSOC_ scores of both HGSOC groups were higher than the non‐cancer group's average (Figure 5D and Figure S11, Supporting Information), and the difference was significant (*P* = 0.0002 for non‐cancer versus early stage; *P* = 0.0004 for non‐cancer versus late stage). However, there was no significant difference in the EV_HGSOC_ scores between the two HGSOC groups (*P* = 0.949; Tukey's multiple comparisons tests).

We next explored whether the nine HGSOC markers can simultaneously classify the three groups (i.e., non‐cancer, early‐stage HGSOC, and late‐stage HGSOC). We applied linear discriminant analysis (LDA) for this multi‐class separation while varying the number of markers used as predictors. The five markers (EpCAM, CD24, HE4, VCAN, TNC) from HGSOC diagnostics were selected again (Table S2, Supporting Information), achieving the highest classification accuracy while minimizing the number of markers. The overall accuracy of the three‐group classification was 0.75 with a 95% confidence interval of (0.63, 0.89). Importantly, the five‐marker model differentiated the early‐stage HGSOC group from both late‐stage HGSOC and non‐cancer groups (Figure 5E and Table S3, Supporting Information) with a specificity of 0.91 ( = 31/34) and a sensitivity of 0.76 ( = 13/17). We further validated our EV results by examining the tumor tissues of HGSOC patients (*n* = 8). Both STIC lesions and early HGSOCs stained positive for the five markers, with strong expression in both STIC and HGSOC (Figure 5F and Figure S12, Supporting Information).

## 3 Discussion

The success of nascent or future molecular tests for early cancer detection will require high standards to be met or exceeded. Putative biomarkers should be highly specific to minimize false positives, sensing modalities should be tuned to catch weak signals from small tumors, and test procedures/assays should be scalable and minimally invasive for translation into a population‐wide screening. The current study was designed to systematically address these challenges, with a focus on HGSOC, the most common and lethal OvCa subtype. We reasoned that EVs could be a potent analytic target since they are produced by expanding tumor cells and are readily accessible in peripheral blood. We thus established an EV‐based HGSOC test by i) developing a high‐throughput EV screening platform (SAViA) and ii) defining HGSOC molecular markers by analyzing EVs derived from oncogenic murine FT cells. Our longitudinal study showed that HGSOC marker expression increased in circulating EVs when HGSOC was initiated in an orthotopic murine model. To our knowledge, this is the first study to examine fallopian tube‐derived EVs. A pilot study with human patient samples further supported our preclinical findings; plasma EV analysis enabled us to identify HGSOC cases (AUC of 0.97 for HGSOC versus non‐cancer) and differentiate early‐stage from advanced‐stage cancers. These findings raise the promise of non‐invasive surveillance protocols for women at high risk of developing the disease, including BRCA mutation carriers.

Our marker selection was guided by emerging insights that HGSOC may arise from precursor lesions within the fallopian tube. We used transformed mFT cells with a dominant negative *Tp53^R172H^* mutation, loss of *Pten*, and varying *Brca* deletions. Alterations in these genes are recognized as the earliest changes seen in fallopian tube STICs and a subset of HGSOCs.^[^ ^5^ ^]^ An average woman's lifetime risk of developing OvCa is 1 in 78. However, this risk increases to nearly 39% in women carrying *BRCA1* mutations and up to 11% in women with *BRCA2* mutations. *TP53* mutation was found in more than 95% of HGSOC patients.^[^ ^50^, ^51^ ^]^ Complete and partial loss of *PTEN* was about 15% and 50%–60%, respectively, in HGSOC cases.^[^ ^52^ ^]^ The modified mFT cells harbored these common genotype variations. We selected candidate targets from mFT‐EV proteomes and exploited a mouse model that recapitulated tumor initiation and progression. The EV signature of HGSOC was then identified by analyzing serial blood samples from these animals. Of note, mFT cells can also be used to model tumor metastasis and test new therapeutics, thus supporting their additional scientific and clinical potential impact^[^ ^32^, ^53^, ^54^, ^55^, ^56^, ^57^ ^]^

Future research should interrogate EVs’ diagnostic impact for monitoring precursor evolution before or following risk reduction surgery (in high‐risk contexts) or the chance encounter with an isolated STIC. First, there is a need to increase patient cohorts across the entire spectrum of the disease (i.e., benign, early, advanced stages, as well as the other more rare ovarian cancer subtypes). Such specimens will be crucial in validating the marker panel for early detection and can be used to explore which EV signatures are retained and which are specific to subtypes. We do note that our patient population reflects a single institution experience. Acquiring patient samples from multiple institutions would further enable us to account for ethnic and geographical diversities. Second, we could include other EV‐associated cargo in the diagnostic algorithms. For instance, analyzing EV RNAs may boost current detection sensitivity through enzymatic target amplification and can provide complementary molecular traits (e.g., gene mutation). Herein, EV protein results could be exploited to immuno‐capture early‐stage OvCa EVs, thereby enhancing diagnostic specificity. Third, we can expand marker panels to obtain diverse tumor information. Previous studies have shown that EV molecular profiles can be correlated with OvCa drug resistance, poor prognostics, and metastatic propensities.^[^ ^58^, ^59^ ^]^ The orthotopic mFT mouse model could be an excellent translational test bed to corroborate such findings and refine markers, as it can mimic tumor initiation, progression, and spread into the peritoneal cavity. Moreover, mFT mouse models bearing various genetic subtypes have shown a potential for genotype‐specific monitoring of tumor progression and treatment responses.^[^ ^60^ ^]^ These advances should deepen our insight into OvCa's origin and early evolution and eventually improve patient outcomes through informed cancer management. In particular, with an efficient surveillance test in place, young women at high risk could choose a stepwise risk‐reduction surgery (salpingectomy first followed by oophorectomy closer to natural menopause), minimizing the side effect of early surgical menopause.

## 4 Experimental Section

### SAViA Assay

We suspended EV isolates in 0.05 m carbonate‐bicarbonate buffer (pH 9.4; Millipore Sigma, C3041) and loaded the solution into a high‐binding 384‐well plate (Greiner, 781 077). Under this pH, proteins tend to be deprotonated and attain high solubility, which improves their adsorption to the plate surface. The sample volume (25 µL per well) was the nominal amount recommended for a 384‐well plate. The plate was placed on an orbital shaker, and EVs were allowed to adsorb to the plate surface (8 h, 4 °C). Following the incubation, we washed the plate with PBS buffer containing 0.1% Tween 20 (0.1% PBS‐Tw) two times and treated it with a blocking buffer (2% BSA in PBS) for 1 h at RT. After washing the plate with 0.1% PBS‐Tw, primary antibodies (1% BSA in 0.1% PBS‐Tw) were added and allowed them to react (1 h, RT). Excess antibodies were removed via triple‐washing with 0.1% PBS‐Tw. Subsequently, EVs were similarly labeled with biotinylated secondary antibodies (1% BSA in 0.1% PBS‐Tw). We next reacted samples with streptavidin‐HRP (ThermoFisher, 21 130; 1% BSA in 0.1% PBS‐Tw) for 30 min at RT, triple‐washed them with 0.1% PBS‐Tw, and performed tyramide signal amplification by adding 6 µg mL^−1^ tyramide‐biotin (Millipore Sigma, SML2135) in amplification buffer (0.003% Hydrogen peroxide in 0.1× borate buffer). After a 30‐min incubation at RT, we triple‐washed samples with 0.1% PBS‐Tw and labeled them with streptavidin‐BV510 (Biolegend, 405 234; 1% BSA in 0.1% PBS‐Tw). After a 30‐min incubation, we washed samples (0.1% PBS‐Tw) and then measured their fluorescence intensities with a plate reader (Tecan) at 405/490 nm excitation/emission wavelengths with 20 nm bandwidth.

### Conventional ELISA

We diluted CD63 antibodies (Ancell, 215‐020) in PBS (4 µg mL^−1^) and loaded the solution (50 µL well^−1^) onto a 96 well‐plate (Nunc MaxiSorp flat‐bottom, ThermoFisher, 44‐2404‐21). After incubation at 4 °C for 8 h, the plate was washed twice with 0.1% PBS‐Tw and treated with a blocking buffer (2% BSA in PBS) for 1 h at RT. For EV capture, we washed the plate twice with 0.1% PBS‐Tw, added EV samples (50 µL well^−1^), and incubated them for 1 h at RT. Biotinylated CD63 antibodies (BioLegend, 353 018) were then loaded (500 ng mL^−1^; 50 µL well^−1^) to react for 1 h at RT, and the excess antibodies were removed. For the signal generation, we first loaded streptavidin‐HRP (diluted 1:20 000 in 0.1% BSA; BioLegend, 405 210; 50 µL well^−1^) and incubated the mixture for 20 min at RT. After triple‐washing the plate with PBS, we added 100 µL of 3,3′,5,5′‐tetramethylbenzidine (TMB, BioLegend, 421 101) per well and incubated the mixture for 30 min at RT. We stopped the reaction by adding 50 µL of stop solution and measured the absorbance at 450 nm on a plate reader (Tecan).

### mFT Cell Generation

Murine fallopian tube cells were isolated from *Brca/Tp53/Pten* GEMMs of the following four genotypes: mFT3707 (*Brca1^+/−^*, *Tp53^mut^*, *Pten^−/−^*), mFT3635 (*Brca1^−/−^*, *Tp53^mut^*, *Pten^−/−^*), mFT3665 (*Brca2^+/−^*, *Tp53^mut^*, *Pten^−/−^*), and mFT3666 (*Brca2^−/−^*, *Tp53^mut^*, *Pten^−/−^*). The fallopian tubes were extracted, digested for 48 h in a solution of 25 mL of Minimum Essential Media supplemented with 35 mg of Pronase and 2.5 mg of DNase, and then plated in a 96‐well plate. After ≈2 weeks, at which point cell death became apparent, 1 µg mL^−1^ doxycycline hyclate (Sigma‐Aldrich, D9891) was added to the media to trigger Cre‐mediated recombination of the genes of interest under the control of the PAX8 promoter. Cells were initially propagated in 96‐well plates until they were transferred to a 10 cm plate. For most cell lines, this process took roughly 10 passages and 2 months. The mFT culture media consisted of equal parts DMEM:F12 and M199 supplemented with HEPES pH 7.4 (10 mm), glutamine (2 mM), EGF (10 ng mL^−1^), ITS‐A (10 µg mL^−1^), Bovine Pancreas Insulin (10 µg mL^−1^), Hydrocortisone (0.5 µg mL^−1^), Cholera Toxin (25 ng mL^−1^), Retinoic Acid (25 ng mL^−1^), BSA (1.25 mg mL^−1^), Heat‐inactivated FBS (0.75% by volume), and FBS (1% by volume).

### Luciferization of Cells

mFT cells were plated and grown to 75% confluency in a 6‐well plate. 0.89 µg VSV‐G plasmid (Addgene, 8454), 1.38 µg Delta 8.9 plasmid, and 1.78 µg of a luc/mCherry lentiviral construct were added to 250 µL of Opti‐MEM (Invitrogen, 31 985 070). In a separate vial, 10 µL of Lipofectamine 2000 (Invitrogen, 11 668 019) was added to 250 µL of Opti‐MEM and incubated for 5 min at room temperature (RT). After incubation, the lentiviral mixture was combined with the Lipofectamine solution and incubated at RT for 20 min. This mixture was then added to one of the wells and incubated for 24 h at 37 °C. The following day, the media was replaced with antibiotic‐free DMEM and incubated at 37 °C for another 24 h. On the fourth day, the media was collected and centrifuged at 1000 rpm for 10 min, and the supernatant was stored before refreshing the media in the plate again with antibiotic‐free DMEM. The media in the other 5 wells of the plate was aspirated and replaced with 600 µL of the collected supernatant from the transfected well in addition to 8 µg mL^−1^ of polybrene and 1.4 mL of antibiotic‐free DMEM. The same process was repeated the following day. After this procedure, the cells were sorted for mCherry expression at the Brigham & Women's Flow Cytometry Core.

### Characterization of mFT Cells

To analyze cell genes, we isolated genomic DNA using a Gentra Puregene Cell Kit (Qiagen, 158 767) according to the manufacturer's protocol. DNA was used to analyze recombination via polymerase chain reaction (PCR), as previously described.^[^ ^32^ ^]^ For the *Brca1* PCR, 100 ng of DNA was used for the positive control, while 500 ng was used for the heterozygous and homozygous deletion samples. For the *Brca2* PCR, 100 ng of DNA was used for the control and the heterozygote, while 1000 ng of DNA was used for the homozygous deletion sample. For *Tp53* and *Pten* PCR, we used 300 ng of DNA samples. All gel electrophoresis was completed using a 1517–100 bp ladder (BioLabs, N3231L). To analyze protein cellular protein, we performed western blots for CA125 (Abbiotec, 250 556), γ‐H2AX (CST, 9718S), PAX8 (Proteintech, 10 336), and β‐actin (Sigma, A2228), as previously described.^[^ ^32^ ^]^ Western blots were completed with a 250–10 kD Protein Precision Plus Kaleidoscope Standard (BioRad, 1 610 375). Gel images were taken with the ChemiDoc XRS+ System (BioRad, 1 708 265).

### Immunofluorescent Cell Imaging

We placed glass coverslips in a 6‐well culture plate and soaked them with 70% ethanol for 1 h. After washing the coverslips with PBS, we seeded about 3 × 10^5^ cells per well and cultured them overnight in culture media at 37 °C. The cells were then washed with PBS and fixed with 4% paraformaldehyde in PBS for 20 min at RT. The slips were then rinsed with PBS, followed by permeabilization with 0.5% Triton X‐100 in PBS (PBS‐T; 30 min, RT). The cells were rinsed again with PBS, and the coverslips were moved to a paraffin‐covered plate that functioned as an incubation chamber. The cells were blocked with 1% BSA in 0.05% PBS‐T for 1 h at RT, using ≈250 µL of this blocking solution per coverslip. The blocking solution was aspirated, and the cells were incubated with 200–300 µL of CA125 (Abbiotec, 250 556), p53 (Leica Biosystems, CM5), Ki67 (Novus Bio, NB110‐89719), or WT1 (Abcam, ab15249) antibody solution diluted in the aforementioned blocking buffer. After incubation (1 h, RT), cells were triple‐washed with 0.05% PBS‐T in 5‐min intervals. Finally, cells were incubated with fluorophore‐conjugated secondary antibodies (1 h, RT) and then triple‐washed with 0.05% PBS‐T in 5‐min intervals. The coverslips were mounted onto slides using Fluoromount‐G, allowed to dry, sealed, and imaged using an EVOS FL Auto 2 microscope. We used ImageJ (version 10.2) to adjust for background fluorescence.

### Spheroid Formation

About 6.5 × 10^3^ cells per well were seeded in a 96‐well ultra‐low adhesion plate (Corning, CLS3474). The cells were imaged on days 3, 7, and 14. For spheroid‐to‐monolayer experiments, 6 × 10^5^ cells were seeded per well in a 6‐well ultra‐low adhesion plate (Corning, CLS3471) before being plated in a 10 cm dish.

### EV Proteomics

We plated an equal number of mFT cells (mFT3635, mFT3666) in a 150 cm^2^ culture dish. When cells reached about 99% confluency, they were rinsed with PBS and allowed to grow in a serum‐depleted culture medium (48 h). We next collected cell culture media and removed cell debris via centrifugation (10 000×g, 3 min). To collect EVs, we first concentrated supernatants using a centrifugal filter (Centricon Plus‐70, Millipore Sigma) and loaded a concentrated media (0.5 mL) to a qEV column (IZON, SP1). EV fractions (F7‐F9) were collected (1.5 mL). Total protein concentrations were measured via a Qubit protein assay (ThermoFisher, Q33212). For proteome profiling, isolated EVs were sent to an external vendor (BGI company) that performed EV lysis, protein recovery, sample digestion, and nano‐flow LC‐MS/MS analysis. Abundant proteins were selected by the spectral counting method provided by BGI.

### Single EV Imaging

We diluted plasma EVs in PBS and removed aggregated using a 0.22‐µm Millex‐GV syringe filter (Millipore Sigma, SLGV004SL). Filtered EVs were captured on a glass slide (Electron Microscopy Sciences). Following a 30‐min incubation at RT, the slide was twice washed with PBS. After incubation with a mixture of a fixation buffer (4% formaldehyde) and a permeabilization buffer (BD Biosciences, 554 723), EV samples were treated with Superblock (ThermoFisher, 37 518) for 1 h and labeled (90 min, RT) with a mixture of primary antibodies against CD63, CD9, PAX8, and one of the mFT markers (CA125, VCAN, TNC, FOLR1, HE4). IgG antibody was used as a control. Samples were then triple‐washed with 0.01% PBS‐Tw (PBS buffer containing 0.01% Tween 20), labeled (30 min, RT) with fluorophore‐conjugated secondary antibodies, and triple‐washed again with PBS‐Tw. Finally, fluorescence images were taken by a Nikon A1R confocal microscope with a 60× objective. All the acquisition settings (i.e., objective, image size, laser power, gain) were kept the same. Images were processed (i.e., background subtraction) and analyzed with Comdet v0.5.5 in Image J software (NIH).

### Tumor Mouse Model

Animal studies were conducted following the guidelines provided by the Institutional Animal Care and Use Committee (IACUC) of Brigham and Women's Hospital, Harvard Medical School (Protocol 2018N000216), and the Whitehead Institute at the Massachusetts Institute of Technology (Protocol 1020‐098‐23). For orthotopic injections, we anesthetized 12–16 weeks old NOD.Cg‐*Prkdc^scid^ Il2rg^tm1Wjl^/*SzJ (NOD‐*scid* IL2Rgamma^null^) female mice (Whitehead Institute) with 1.25% Avertin via intraperitoneal injection (400 mg kg^−1^). Using aseptic techniques, we made a 1 cm incision along the thoracic region between the third and fourth mammary gland (roughly 0.5 cm from the spine) to expose the peritoneum and another small incision (0.5 cm in length) into the peritoneal wall above the ovary and ovarian fat depot. The adipose tissue and ovary were pulled through the peritoneal incision to expose 0.5 cm of adipose tissue. We injected about 10^6^ mFT cells (resuspended in 10–20 µL of a 1:1 dilution of Matrigel in PBS) into the fallopian tube using a Hamilton syringe. Once the procedure was complete, we carefully pushed back the adipose depot through the peritoneal wall and closed the peritoneum using sterile, biodegradable J204G 4‐0 Vicryl violet sutures (Ethicon). After suturing the peritoneum, we gathered the skin and closed the incision with wound clips. Mice were kept warm on a heating pad until fully recovered. Meloxicam was administered intraperitoneally (5 mg kg^−1^) immediately after surgery and 24 h later. For intraperitoneal injections, 6‐week‐old NOD.CB17‐Prkdcscid/NCrCrl (SCID) mice (Charles River Laboratories, Strain #394) were injected intraperitoneally with 5 × 10^6^ cells resuspended in PBS. For bioluminescence imaging, mice were injected (IP) with luciferin (25% of their body weight) in PBS. After 4 min, isoflurane was administered to anesthetize the mice for imaging on the Xenogen IVIS‐100 Imaging System.

### Serial EV Monitoring from Tumor‐Bearing Mice

About 200 µL of blood was sampled from the retroorbital sinus of a mouse using a heparinized microhematocrit tube. Blood samples were immediately centrifuged at 2000×g for 15 min at 4 °C, plasma supernatant was collected, and plasma samples were stored at −80 °C. To isolate EVs, about 50 µL of plasmas were processed with qEV columns (IZON, SP2) per the manufacturer's instruction.

### Human Samples

The study protocol was reviewed and approved by the Institutional Review Board of Dana–Farber/Harvard Cancer Center (IRB number 07–049). On a rolling basis, we recruited patients who met the eligibility criteria (ovarian cancer diagnosis based on tissue biopsy) and gave informed consent. Plasma samples were centrifuged at 2000×g for 3 min to remove cell debris, and supernatants were collected. About 100 µL of plasmas were used for EV isolation through qEV columns (IZON, SP2). All clinical samples were measured at least three times (technical replica) for each protein marker, and the mean values were used for analyses. All attempts at replication were successful. Blinding was not performed as the data analysis was not subjective. The sample number was powered to determine whether the EV‐based test offers high diagnostic accuracy. We used area under the ROC curve (AUC) as an accuracy index. The null hypothesis was that the EV test has an AUC of 0.7 (a fair test), whereas the alternative hypothesis was that the EV test performs significantly better (AUC = 0.9, a highly accurate test). The sample size that was used (34 cancer patients and 17 controls) achieves >85% power to detect this AUC difference of 0.2 at a significance level of 5% (two‐sided *z*‐test).

### Immunohistochemistry

Tumors and other organs (ovary, fallopian tube, uterus) were collected from mice for immunohistochemistry (IHC). IHC samples were sent to the Specialized Histopathology Core at Harvard Medical School for staining. Additionally, some IHC experiments were performed at the Whitehead Institute, Massachusetts Institute of Technology. For ascites IHC samples, ascites were collected from the mice, spun down, and cultured under the same conditions as their mFT cell line origins. After the ascites lines were established, we collected about 2 × 10^7^ cells via centrifugation, fixed the cells in 10% formalin, and embedded them in paraffin. The cells were then sectioned and adhered to slides and stained by the Specialized Histopathology Core at Harvard Medical School. Slides were stained for PAX8 (ProteinTech, 10 336), p53 (Abcam, 1431), STMN1 (CST, 13 655), and WT1 (Sigma, 348M‐9).

### Biomarker IHC Validation

The study protocol was reviewed and approved by the Institutional Review Board of Massachusetts General Hospital (IRB protocol, 2022P001059). Formallin‐fixed paraffin‐embedded sections of human patient samples were baked at 56 °C for 30 min and deparaffinized with 10‐min xylene washes (two times). Samples were then rehydrated with 5‐min ethanol washes at increasing dilutions (100%, 100%, 95%, and 75%). Endogenous peroxidase was then blocked using a 15‐min incubation in a 1:1 solution of 100% ethanol and 3% hydrogen peroxide. Antigen retrieval was performed by boiling samples in citrate buffer (Dako, S1699) for 15 min at 110 °C using a NxGen Decloaking Chamber (Biocare, DC2012). Slides were then washed with PBS and blocked with 10% goat serum for an hour at RT. After blocking with serum, the samples were blocked for endogenous biotin and avidin binding sites following manufacturer instructions (Vector, SP‐2001). Sections were then incubated overnight at 4 °C with their respective primary antibodies diluted in PBS. The following day, samples were washed in tris‐buffered saline supplemented with 0.05% tween‐20 (TBS‐Tw) prior to incubating with biotin‐conjugated secondary antibodies for 1 h at RT. Slides were then washed again with TBS‐Tw and incubated for an additional hour at RT with HRP‐conjugated streptavidin (Vector, SA‐5004; 1:100). After a final set of TBS‐Tw washes, slides were visualized using DAB, counterstained with hematoxylin, air‐dried overnight, and coverslips were mounted using Permount mounting medium (Fisher SP15‐500).

### Statistical Analysis

Statistical analysis was performed using GraphPad Prism version 9.4 (GraphPad Software Inc.) or R version 4.1.0. The main text and the figure legends describe data presentation, sample sizes, and statistical methods for specific experiments. For all statistical tests, *P* values < 0.05 were considered significant. i) Marker selection for HGSOC detection. The lasso regression was applied to select candidate markers from the EV profiling data. Specifically, we calculated the cross‐validation error (CVE) and determined the tuning parameter (λ) that minimized CVE. We used the glmnet package in R. ii) HGSOC diagnosis. We assessed the diagnostic ability of EV markers by conducting a ROC analysis. Individual EV markers and their combinations were considered. For each biomarker combination, optimal weights were determined via logistic regression. We constructed ROC curves and determined level cutoffs that maximized the sum of sensitivity and specificity. Standard formulas were used to define sensitivity (true positive rate), specificity (true negative rate), and accuracy [(true positive + true negative)/(positive + negative)]. AUCs were compared following Delong's method. EV_HGSOC_ had the highest AUC value with the minimal number of EV markers used. Analyses were performed using the pROC package in R. iii) Linear discriminant analysis (LDA). An LDA model was constructed to classify clinical samples into three groups: non‐cancer, early‐stage HGSOC (stages I & II), and late‐stage HGSOC (stages III & IV). We varied the number of EV markers used as predictors and constructed a confusion matrix. The five‐marker (EpCAM, CD24, HE4, VCAN, TNC) LDA model showed the highest classification accuracy with the following linear discriminants: LD1 = 0.165·EpCAM + 0.638·CD24 – 0.191·HE4 + 0.172·VCAN + 1.126·TNC; LD2 = 0.268·EpCAM + 0.175·CD24 + 1.106·HE4 + 0.495·VCAN – 0.922·TNC. Confidence intervals for the accuracy of each model were estimated using the bootstrap method with 1000 replicates. We used the MASS and the Boot packages in R.

### Antibodies

All antibodies with their associated dilution and application can be found in Tables S4–S6, Supporting Information.

## Conflict of Interest

The authors declare no conflict of interest.

## Supporting information

Supporting Information

## Data Availability

The data that support the findings of this study are openly available in MassIVE at https://www.MSV000090942@massive.ucsd.edu, reference number 90942.
