# Supplementary material for: Inaugurating High‐Throughput Profiling of Extracellular Vesicles for Earlier Ovarian Cancer Detection
Source: Adv Sci (Weinh). 2023 Jul 23;10(27):2301930. doi: 10.1002/advs.202301930 (PMC10520636; doi:10.1002/advs.202301930)
Supplement: Supplementary file 1 — Supporting Information [file ADVS-10-2301930-s001.pdf]

ADVANCED SCIENCE
Open Access

Supporting Information

for *Adv. Sci.*, DOI 10.1002/advs.202301930

Inaugurating High-Throughput Profiling of Extracellular Vesicles for Earlier Ovarian Cancer Detection

Ala Jo, Allen Green, Jamie E. Medina, Sonia Iyer, Anders W. Ohman, Eric T. McCarthy, Ferenc Reinhardt, Thomas Gerton, Daniel Demehin, Ranjan Mishra, David L. Kolin, Hui Zheng, Jinwoo Cheon, Christopher P. Crum, Robert A. Weinberg, Bo R. Rueda, Cesar M. Castro, Daniela M. Dinulescu* and Hakho Lee**

SUPPLEMENTARY INFORMATION

Inaugurating high-throughput profiling of extracellular vesicles for earlier ovarian cancer detection

Ala Jo[1,2,3], Allen Green[4], Jamie E. Medina[4#], Sonia Iyer[5#], Anders W. Ohman[4#], Eric T. McCarthy[4#], Ferenc Reinhardt[5], Thomas Gerton[4], Daniel Demehin[4], Ranjan Mishra[5], David L. Kolin[4], Hui Zheng[6], Jinwoo Cheon[3], Christopher P. Crum[4], Robert A. Weinberg[5], Bo R. Rueda[7], Cesar M. Castro[1,8*], Daniela M. Dinulescu[4*], Hakho Lee[1,2,3*]

[1] Center for Systems Biology, Massachusetts General Hospital, Harvard Medical School, Boston, MA 02114, USA

[2] Department of Radiology, Massachusetts General Hospital, Harvard Medical School, Boston, MA 02114, USA

[3] Center for Nanomedicine, Institute for Basic Science, Seoul, 03722, Republic of Korea

[4] Division of Women's and Perinatal Pathology, Department of Pathology, Brigham and Women's Hospital, Harvard Medical School, Boston, MA, 02115, USA

[5] Whitehead Institute, Massachusetts Institute of Technology, Cambridge, MA, 02142, USA

[6] Biostatistics Center, Massachusetts General Hospital, Boston, MA 02114, USA

[7] Division of Gynecologic Oncology, Department of Obstetrics and Gynecology, Massachusetts General Hospital, Boston, MA, 02114, USA

[8] Cancer Center, Massachusetts General Hospital, Harvard Medical School, Boston, MA, 02114, USA

[#]Equal contribution (J.E.M., S.I., A.W.O., E.T.M.)

*Equal contribution, Co-Corresponding Authors (C.M.C., D.M.D., H.L.)

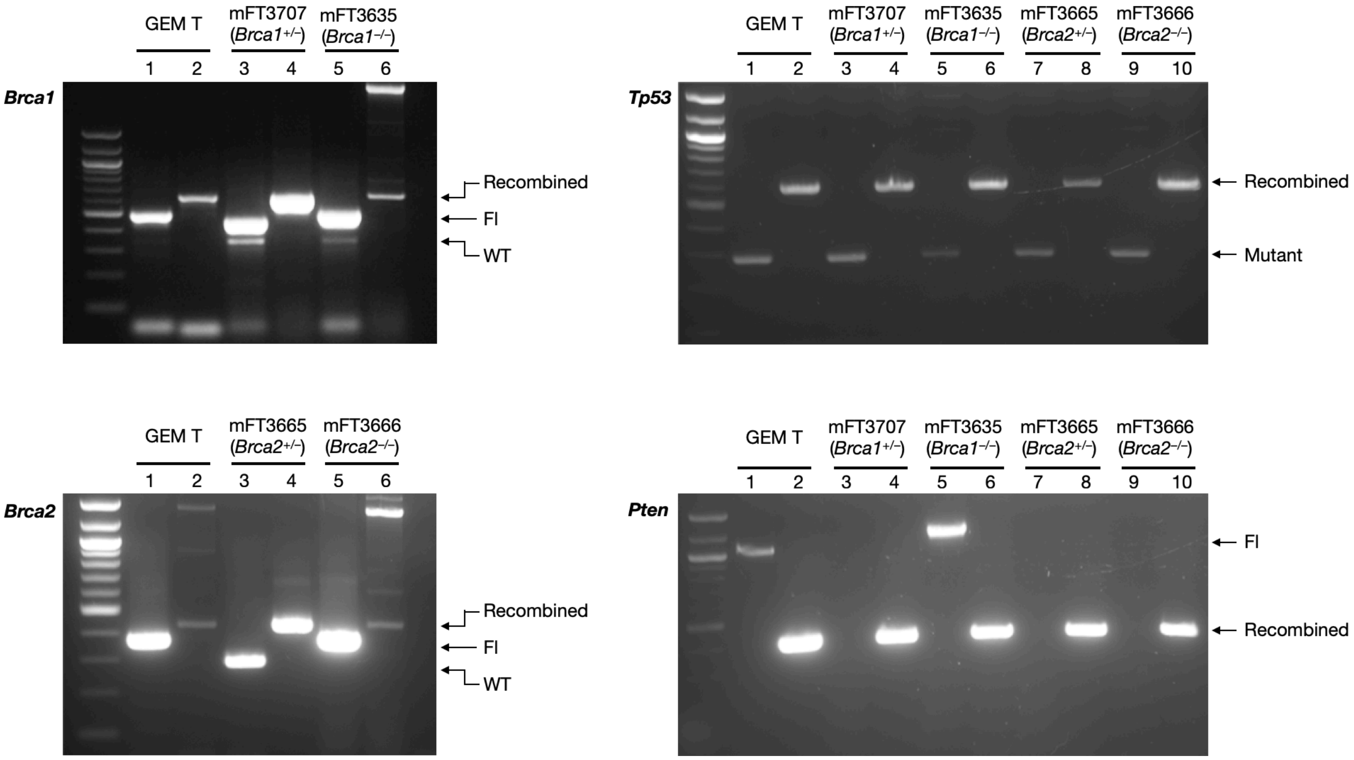

Supplementary Figure S1. Genotyping of mFT cell lines. *Brca1*, *Brca2*, *Tp53*, and *Pten* genes were amplified by a polymerase chain reaction and measured by gel electrophoresis. The following cell lines were characterized: mFT3707 (*Brca1*$^{+/-}$, *Tp53*^{mut}, *Pten*$^{-/-}$), mFT3635 (*Brca1*$^{-/-}$, *Tp53*^{mut}, *Pten*$^{-/-}$), mFT3665 (*Brca2*$^{+/-}$, *Tp53*^{mut}, *Pten*$^{-/-}$) and mFT3666 (*Brca2*$^{-/-}$, *Tp53*^{mut}, *Pten*$^{-/-}$). Odd numbered lanes have unrecombined DNA, and even numbered lanes have *Cre*-mediated recombined DNA. GEM T, tumors from genetically engineered model tumors; Fl, floxed allele; WT, wild-type allele.

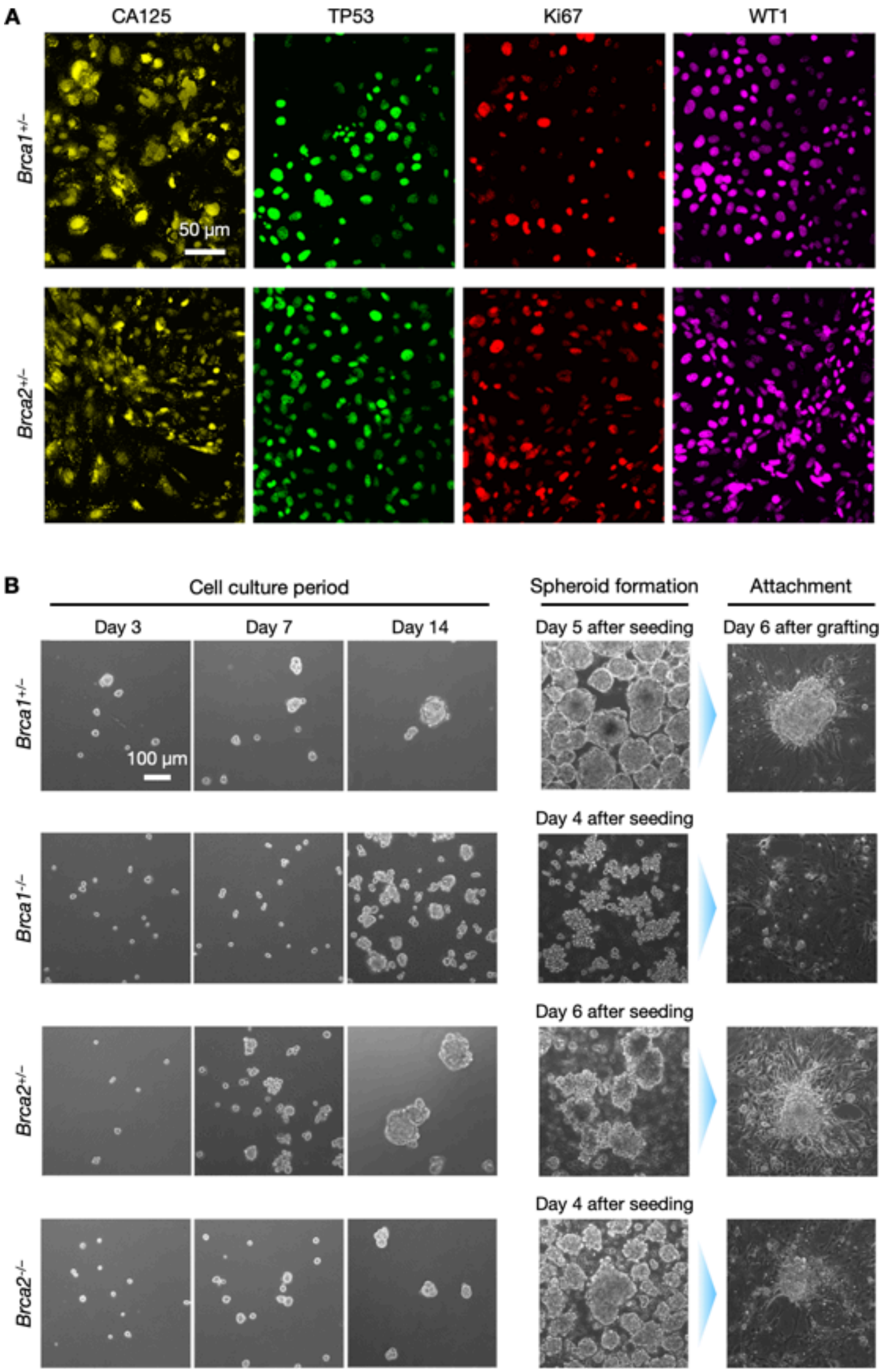

Supplementary Figure S2. *In vitro* Characterization of mFT cell lines. (A) Immunofluorescence staining of CA125, TP53, Ki67, and WT1 in additional mFT cell lines: mFT3707 (*Brca1*$^{+/-}$) and mFT3665 (*Brca2*$^{+/-}$). **(B)** Spheroid formation of mFT cell lines. Microscope images show spheroid formation and growth over a 14-day period. Spheroids were then transferred from ultra-low adhesion plates to attachment plates to demonstrate spheroids' capability to adhere to surfaces.

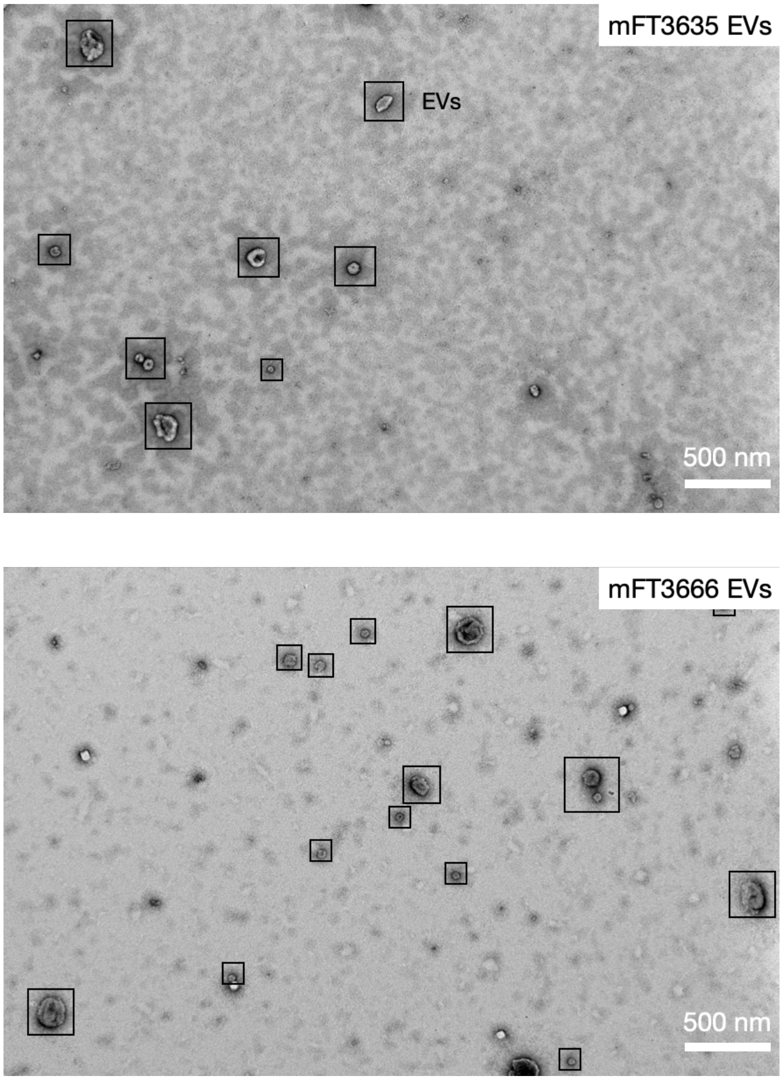

Supplementary Figure S3. Large field-of-view TEM images of mFT EVs. (Top) EVs isolated from mFT3635 cells. (Bottom) EVs isolated from mFT3666 cells. Black squares indicate EVs.

A

Cell line	EV sample replicate	Input [EV] (particles/mL)	Number of proteins identified	Total number of unique proteins
mFT3635 (*Brca1*$^{-/-}$)	1	2.5×10^{11}	571	N_{3535} = 631
	2	9.1×10^{10}	507	
	3	1.7×10^{10}	465	
mFT3666 (*Brca2*$^{-/-}$)	1	1.4×10^{9}	133	N_{3666} = 215
	2	6.3×10^{9}	177	
	3	4.0×10^{8}	157	

B

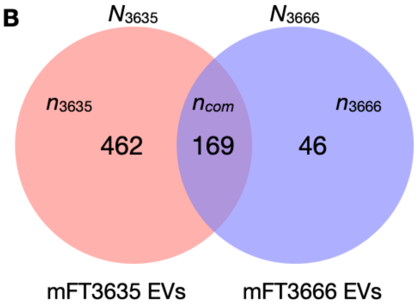

Supplementary Figure S4. Proteome analysis of mFT EVs. (A) Summary characteristics of mFT-EV samples isolated from mFT3635 and mFT3666 cell lines. Three replicates from each cell line were used for the proteome profiling. The input EV amount was larger for mFT3635 than for mFT3666, likely leading to more proteins identified in mFT3635 EV samples. **(B)** The Venn diagram shows the distribution of proteins identified in mFT3635- and mFT3666-EV samples. n_{3635} (n_{3666}) is the number of proteins unique to mFT3635 (mFT3666) EVs, and n_{com} is the number of proteins common to both EV types. The observed n_{3635}/n_{3666} = 462/42 ≈ 10 is attributed to the imbalance in the total proteins detected. Specifically, the total number of proteins is written as $N_{3635} = n_{3635} + n_{com}$ and $N_{3666} = n_{3666} + n_{com}$. From the mFT3635-EV data, n_{com}/N_{3635} = 169/631 = 0.27, which leads to $n_{3635} = 2.7 \cdot n_{com}$ or equivalently $N_{3635} = 3.7 \cdot n_{com}$. Based on the total protein ratio (N_{3635}/N_{3666} = 2.9), $N_{3635} = 3.7 \cdot n_{com} = 2.9 \cdot n_{3666} + 2.9 \cdot n_{com}$, which gives $n_{3635} = 0.27 \cdot n_{com}$. Thus, the expected n_{3635}/n_{3666} is 10, which matches the observed value.

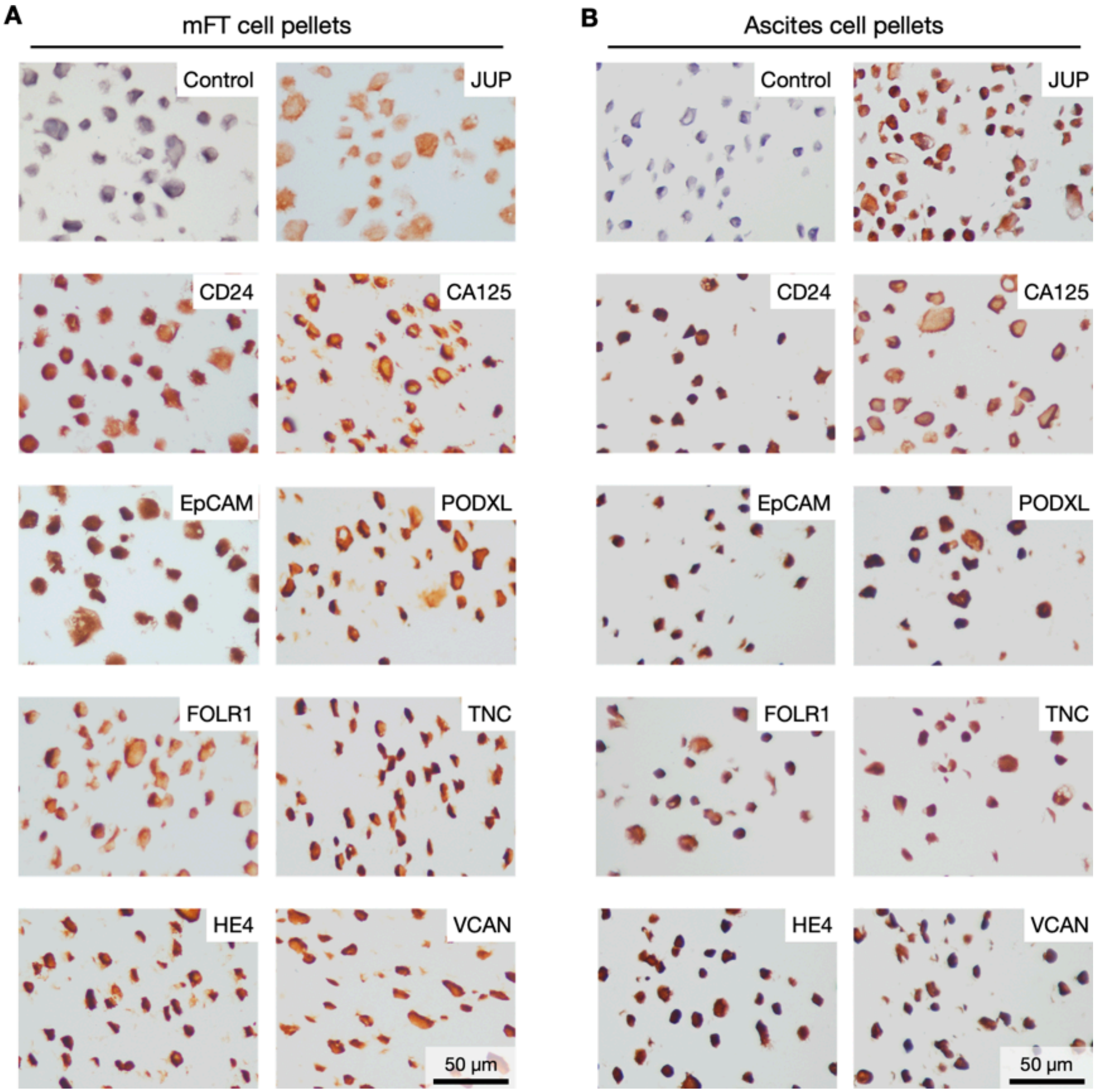

Supplementary Figure S5. Expression of candidate markers in mFT parent and ascites cells. (A) IHC staining of mFT3666 parental cell lines. (**B**) IHC staining of ascites cells collected from a mouse implanted with mFT3666 cells.

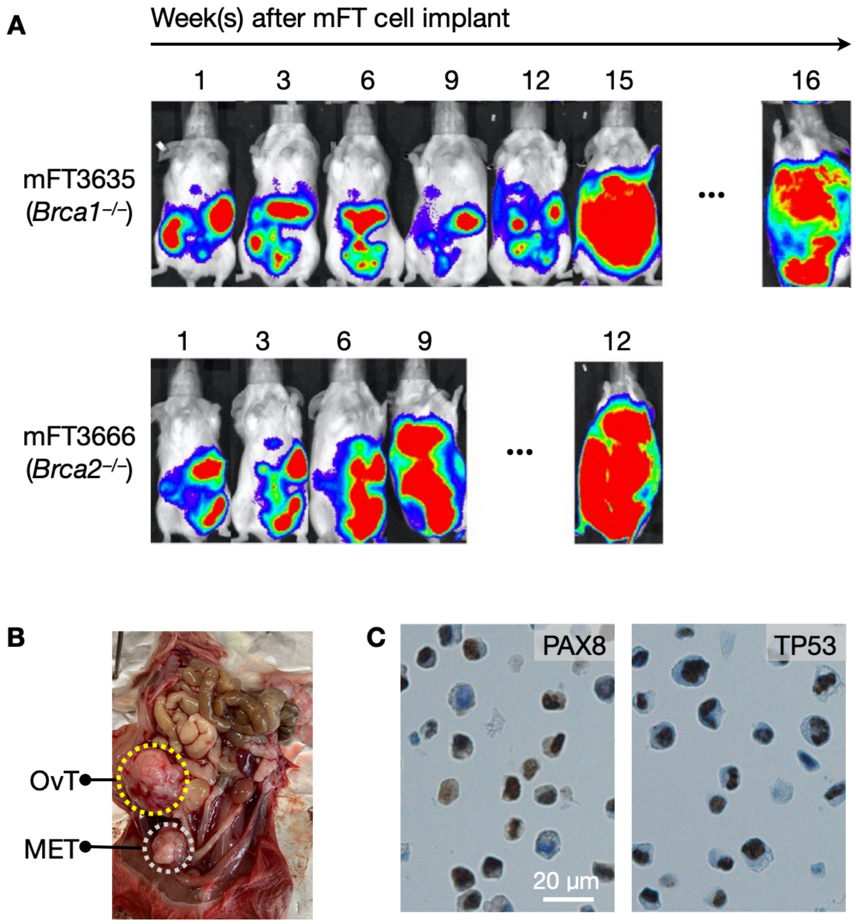

Supplementary Figure S6. Tumorigenic properties of oncogenic mFT cell lines. (A) NOD SCID mice were intraperitoneally implanted with oncogenic mFT cells expressing luciferase. Bioluminescence imaging confirmed the spread of the tumor throughout the peritoneum. **(B)** Presence of tumors in an NSG mouse orthotopically implanted with mFT3666 cells. OvT, primary tumor; MET, metastasis. **(C)** Representative images of ascites cells from mFT3666 engrafted mice. Cells were stained positive for PAX8 and TP53.

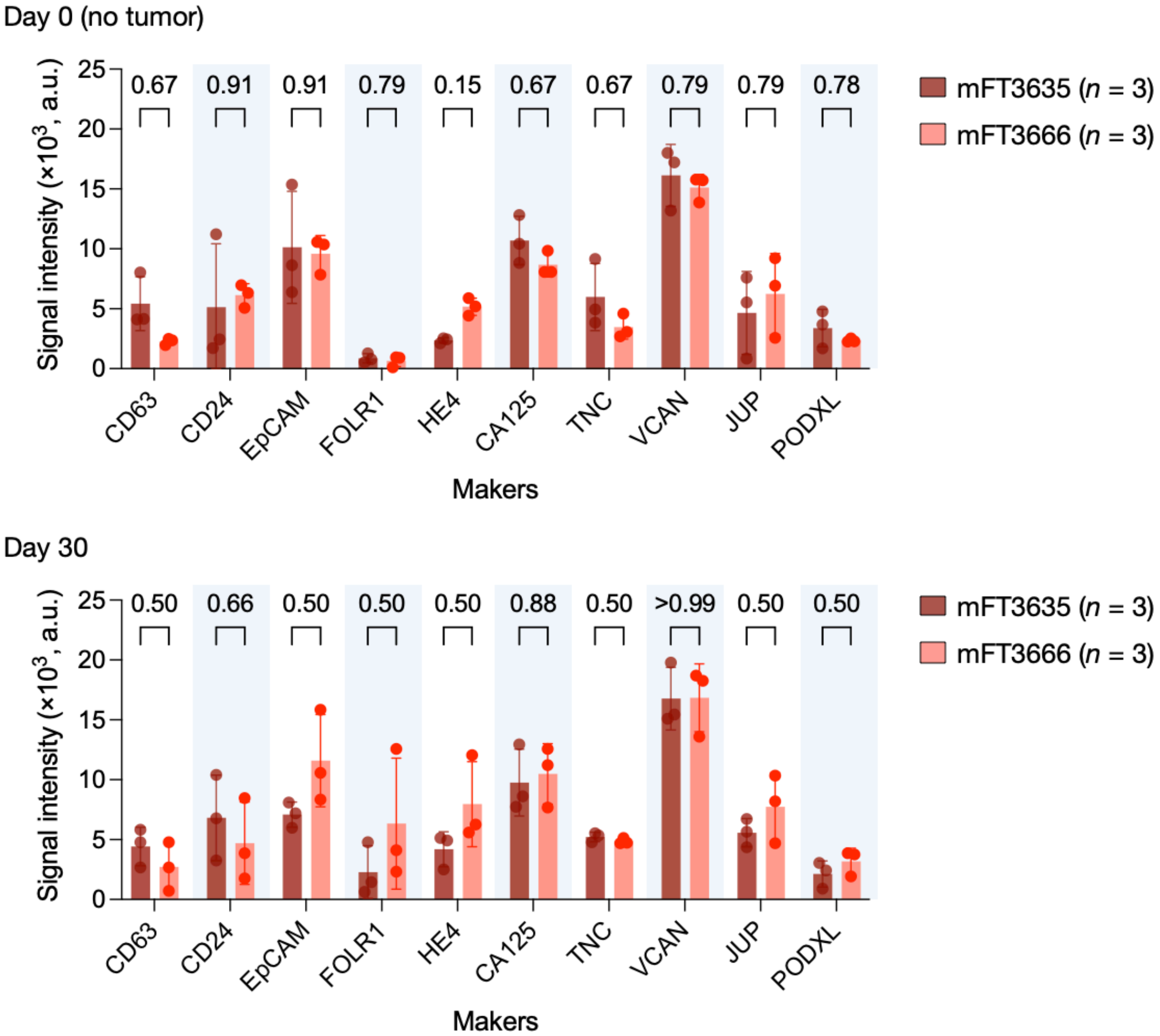

Supplementary Figure S7. Comparison of EV markers between two mouse cohorts. The first cohort (*n* = 3) was implanted with mFT3635 cells, and the other (*n* = 3) with mFT3666 cells. Plasma samples were serially collected before the mFT cell implant (day 0; top) and during tumor growth (day 30; bottom). Circulating EVs were analyzed for a tetraspanin (CD63) and the nine HGSOC markers. No significant differences were observed in marker expression between the two cohorts. Data are displayed as mean ± s.d. Multiple comparison *t*-tests (unpaired, two-sided) were performed with the false discovery rate set <5%. The q value is shown for each marker.

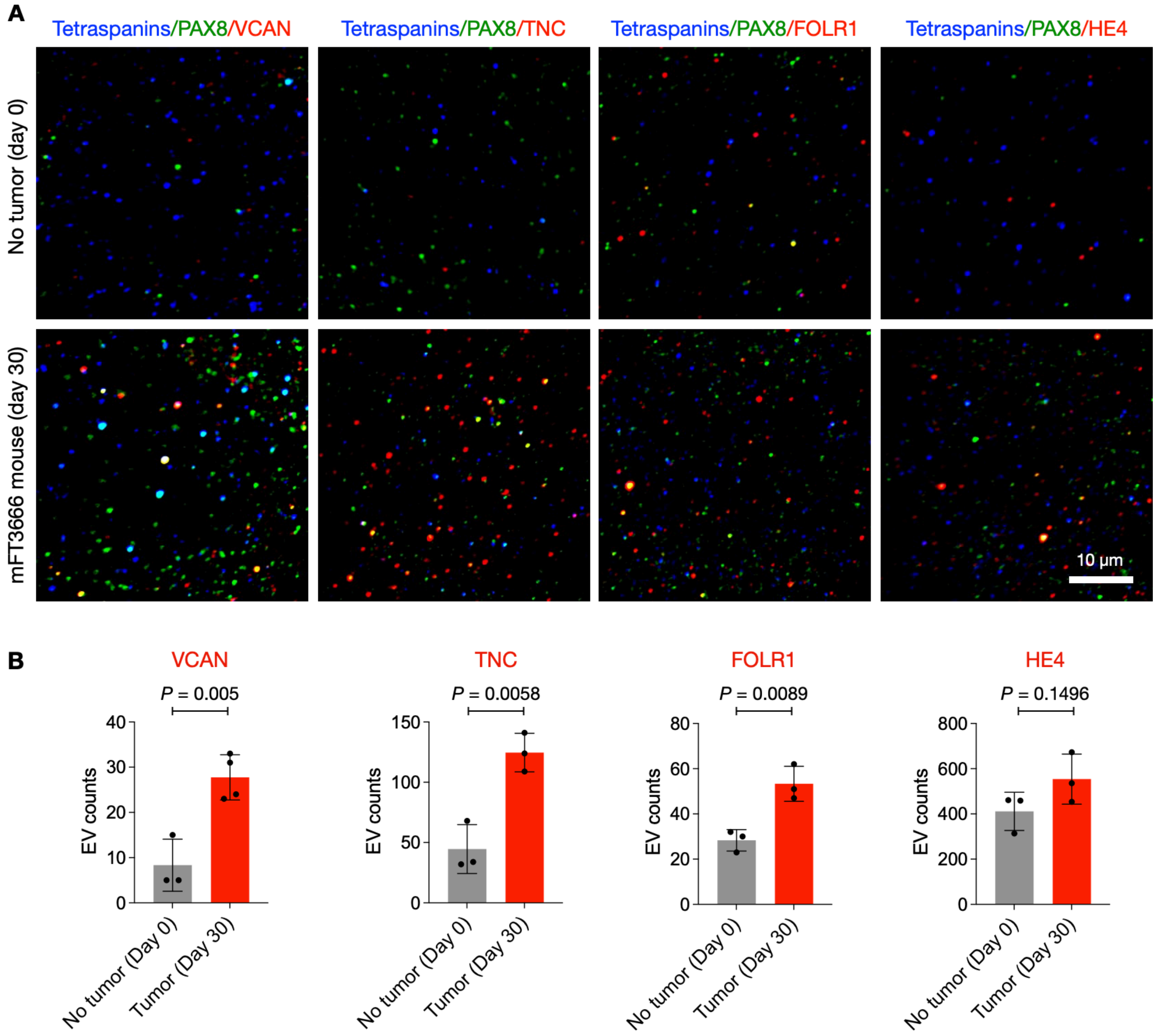

Supplementary Figure S8. Single EV analysis of murine plasma EVs. (A) Plasma samples were obtained from an animal before the mFT cell (*Brca2*$^{-/-}$) implant (day 0) and during tumor growth (day 30). EVs were immunostained for tetraspanins (CD63, CD9, CD81), PAX8 (FT epithelial marker), and HGSOC markers. **(B)** More plasma EVs were counted positive for HGSOC markers in the day-30 sample. Each dot in the graph represents the marker-positive EV number in a given field of view.

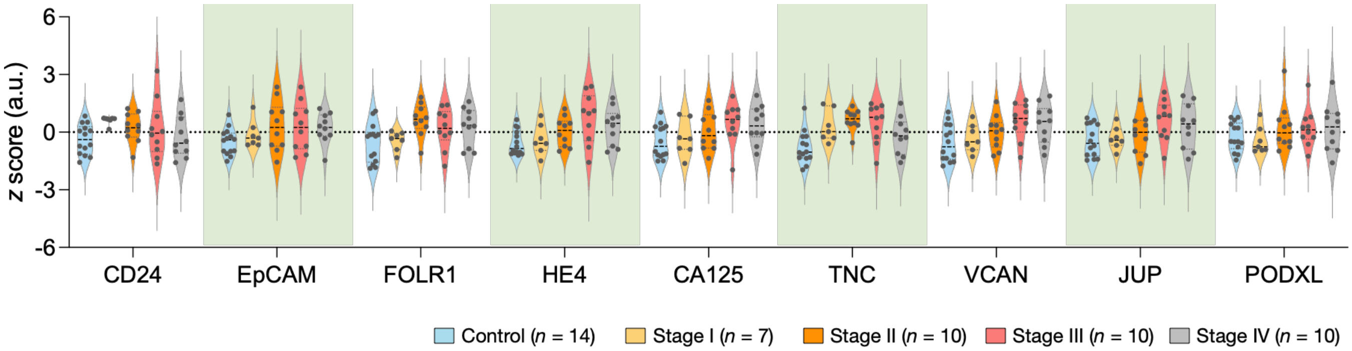

Supplementary Figure S9. Expression levels of nine HGSOC markers. EVs in clinical samples were profiled. For each marker, z-scores were calculated and stratified by the tumor stage.

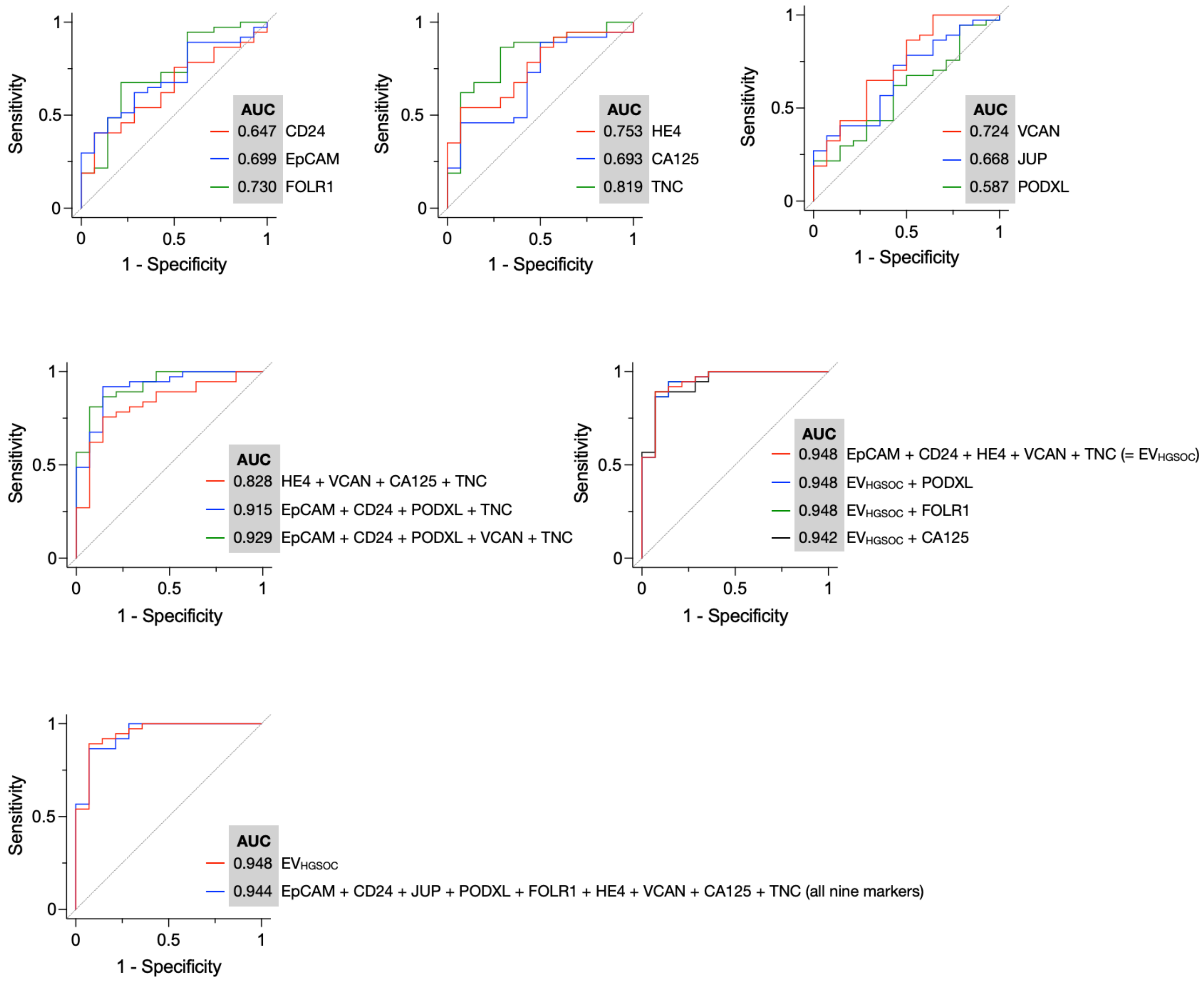

Supplementary Figure S10. ROC analyses for HGSOC detection. Single markers and their combinations were used as classifiers for control (*n* = 14) vs. cancer (*n* = 37) cases. EV_{HGSOC} achieved the highest area under the curve (AUC) with a minimal marker set.

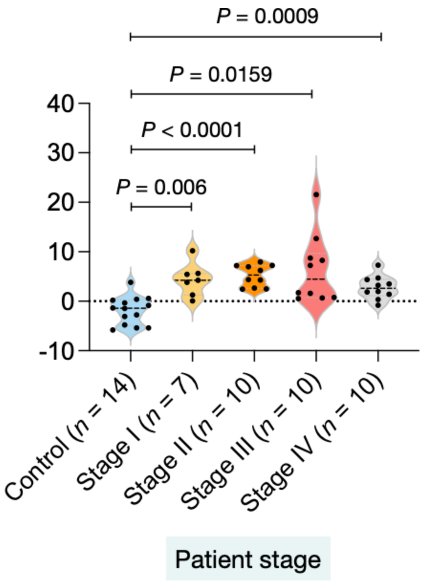

Supplementary Figure S11. Stratified EV_{HGSOC} scores per tumor stage. Adjusted *P* values were obtained from Dunnett's T3 multiple comparisons test.

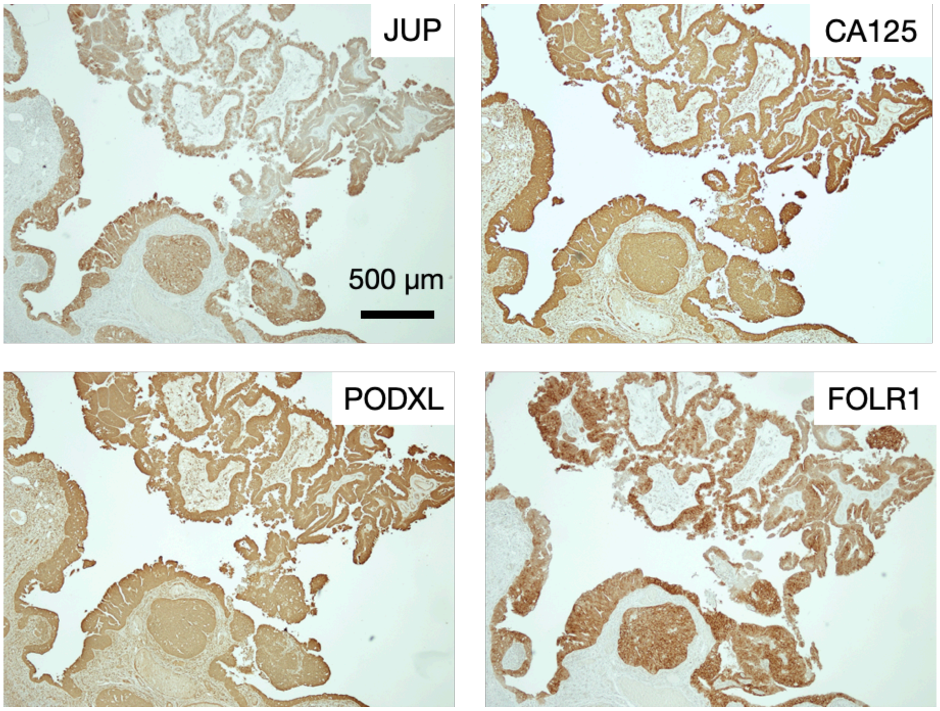

Supplementary Figure S12. Images of IHC stained tissue from an HGSOC patient. STIC and HGSOC were stained positive for JUP, CA125, PODXL, and FOLR1.

Supplementary Table 1. Statistics of HGSOC diagnoses.

Markers	Control vs. early stage				Control vs. all stage			
	AUC	Sen	Spe	Acc	AUC	Sen	Spe	Acc
CD24	0.782	0.965	0.714	0.742	0.647	0.405	0.857	0.549
EpCAM	0.672	0.941	0.429	0.710	0.699	0.487	0.857	0.588
FOLR1	0.714	0.647	0.786	0.710	0.730	0.676	0.786	0.706
HE4	0.693	0.941	0.429	0.710	0.753	0.541	0.929	0.647
CA125	0.626	0.882	0.500	0.710	0.693	0.892	0.500	0.765
TNC	0.882	1.000	0.643	0.839	0.819	0.865	0.714	0.824
VCAN	0.639	1.000	0.357	0.710	0.724	0.865	0.500	0.765
JUP	0.576	0.765	0.500	0.645	0.668	0.730	0.571	0.686
PODXL	0.542	1.000	0.214	0.645	0.587	0.216	100.000	0.431
HE4 + VCAN + CA125 + TNC	0.891	0.765	0.929	0.839	0.828	0.757	0.857	0.765
EpCAM + CD24 + PODXL + TNC	0.971	1.000	0.857	0.935	0.915	0.919	0.857	0.882
EpCAM + CD24 + PODXL + VCAN + TNC	0.979	0.941	0.929	0.935	0.929	0.811	0.929	0.824
EpCAM + CD24 + HE4 + VCAN + TNC (= EV_{HGSOC})†	0.966	0.941	0.929	0.935	0.948	0.892	0.929	0.902
EpCAM + CD24 + HE4 + VCAN + TNC + PODXL	0.966	0.941	0.929	0.935	0.948	0.946	0.857	0.882
EpCAM + CD24 + HE4 + VCAN + TNC + FOLR1	0.966	0.941	0.929	0.935	0.948	0.946	0.857	0.902
EpCAM + CD24 + HE4 + VCAN + TNC + CA125	0.966	0.941	0.929	0.935	0.942	0.892	0.929	0.863
EpCAM + CD24 + JUP + PODXL + FOLR1 + HE4 + VCAN + CA125 + TNC	0.966	0.941	0.929	0.935	0.944	0.865	0.929	0.902

† The minimal marker set was selected for the highest diagnostic accuracy. AUC, area under the curve; Sen, sensitivity; Spe, specificity; Acc, accuracy.

Supplementary Table 2. Performance summary of linear discriminant analysis (LDA).

Markers	Correctly predicted cases				
	Non-cancer (n = 14)	Early (n = 17)	Late (n = 20)	Overall accuracy	95% confidence interval
CD24	2	9	10	0.412	(0.255, 0.628)
EpCAM	6	0	15	0.412	(0.314, 0.608)
FOLR1	6	0	15	0.412	(0.294, 0.588)
HE4	8	6	13	0.529	(0.353, 0.647)
CA125	7	4	17	0.549	(0.353, 0.686)
TNC	9	7	8	0.471	(0.353, 0.647)
VCAN	7	4	15	0.510	(0.353, 0.647)
JUP	5	7	13	0.490	(0.333, 0.628)
PODXL	3	6	15	0.471	(0.294, 0.607)
HE4, VCAN, CA125, TNC	8	11	13	0.627	(0.529, 0.824)
EpCAM, CD24, PODXL, TNC	12	9	10	0.608	(0.529, 0.843)
EpCAM, CD24, PODXL, VCAN, TNC	10	13	13	0.706	(0.588, 0.863)
EpCAM, CD24, HE4, VCAN, TNC	10	13	15	0.745†	(0.628, 0.882)
EpCAM, CD24, HE4, VCAN, TNC, PODXL	10	13	15	0.745	(0.648, 0.902)
EpCAM, CD24, HE4, VCAN, TNC, FOLR1	10	12	15	0.725	(0.628, 0.902)
EpCAM, CD24, HE4, VCAN, TNC CA125	10	12	14	0.706	(0.647, 0.902)
EpCAM, CD24, JUP, PODXL, FOLR1, HE4, VCAN, CA125, TNC	10	14	14	0.745	(0.706, 0.922)

† The minimal marker set for LDA was selected for the highest accuracy in the three-group classification.

Supplementary Table 3. Confusion matrix of the 5-marker LDA model.

		Clinical diagnosis		
		Control	Early HGSOC	Late HGSOC
LDA prediciton	Control	10	0	3
	Early HGSOC	1	13	2
	Late HGSOC	3	4	15

Supplementary Table 4. Primary antibodies used in the study.

Target	Application	Dilution	Vendor	Catalog #
CA125	WB	1:1000	Abbiotec	250556
γ-H2AX	WB	1:1000	CST	9718S
PAX8	WB	1:1000	ProteinTech	10336-1-AP
β-actin	WB	1:2000	Sigma	A2228
CA125	IF	1:250	Abbiotec	250556
p53	IF	1:100	Leica	CM5
KI-67	IF	1:100	Novus	NB110-89719
WT-1	IF	1:300	Abcam	ab15249
PAX8	Murine IHC	1:600	ProteinTech	10336-1-AP
p53	Murine IHC	1:200	Abcam	ab1431
Stathim	Murine IHC	1:2000	CST	13655
WT-1	Murine IHC	1:100	Sigma	348M-9
CD24	Murine IHC	1:50	Bioss	4891R
EpCAM	Murine IHC	1:50	Bioss	1513R
FOLR1	Murine IHC	1:50	Abcam	ab67422
HE4	Murine IHC	1:50	LsBio	LS-C409035
JUP	Murine IHC	1:50	Genetex	GTX114156
CA125	Murine IHC	1:50	LsBio	LS-C408274
PODXL	Murine IHC	1:50	Bioss	1345R
TNC	Murine IHC	1:50	LsBio	LS-C413317
VCAN	Murine IHC	1:50	Bioss	2533R
CD24	Human IHC	1:250	Novus	NBP1-46390
EpCAM	Human IHC	1:100	Bioss	1513R
FOLR1	Human IHC	1:100	Abcam	ab67422
HE4	Human IHC	1:100	LsBio	LS-C409035
JUP	Human IHC	1:100	Genetex	GTX114156
CA125	Human IHC	1:100	LsBio	LS-C408274
PODXL	Human IHC	1:100	Bioss	1345R
TNC	Human IHC	1:100	LsBio	LS-C413317
VCAN	Human IHC	1:100	Novus	NBP1-85432

WB, western blotting; IF, immunofluorescence; IHC, immunohistochemistry.

Supplementary Table 5. Antibodies used for EV ELISA.

Target	Species	Dilution	Vendor	Catalog #	Isotype
CD24	Mouse	1:100	Biolegend	101801	Rat IgG2b
CD24	Human	1:250	Biolegend	311102	Mouse IgG2a
EpCAM	Mouse/Human	1:250, 1:40	Invitrogen	MA5-12436	Mouse IgG1
FOLR1	Mouse/Human	1:500, 1:200	LsBio	LS-C119975	Rabbit IgG polyclonal
HE4	Mouse/Human	1:750, 1:300	LsBio	LS-C409035	Rabbit IgG polyclonal
JUP	Mouse/Human	1:250, 1:100	LsBio	LS-B2204	Goat IgG polyclonal
CA125	Mouse/Human	1:1000, 1:400	LsBio	LS-C408274	Rabbit IgG polyclonal
PODXL	Mouse	1:200	R&D systems	MAB1556	Rat IgG2b
PODXL	Human	1:500	R&D systems	AF1658	Goat IgG polyclonal
TNC	Mouse/Human	1:500, 1:200	LsBio	LS-C413317	Rabbit IgG polyclonal
VCAN	Mouse	1:400	Bioss	BS-2533R	Rabbit IgG polyclonal
VCAN	Human	1:200	Millipore Sigma	HPA004726	Rabbit IgG polyclonal
CD9	Mouse	1:100	Biolegend	124802	Rat IgG2a
CD9	Human	1:250	BD Biosciences	555370	Mouse IgG1
CD63	Mouse	1:200	Biolegend	143902	Rat IgG2a
CD63	Human	1:500	Ancell	215-820	Mouse IgG1
CD81	Mouse	1:200	Biolegend	104901	Armenian Hamster IgG1
TSG101	Mouse	1:200	Genetex	118736	Rabbit IgG polyclonal
Histone 2B	Mouse	1:200	Biolegend	606302	Rat IgG2a
CD63-biotin	Mouse	1:200	Biolegend	143918	-

IgG isotype	Species	Dilution	Vendor	Catalog #
Rat IgG2a	Mouse/Human	*a*	Biolegend	400502
Rat IgG2b	Mouse/Human	*a*	Biolegend	400602
Mouse IgG1	Mouse/Human	*a*	Biolegend	400102
Mouse IgG2a	Mouse/Human	*a*	Biolegend	400202
Rabbit polyclonal IgG	Mouse/Human	*a*	Biolegend	910801
Goat IgG Isotype	Mouse/Human	*a*	Novus Biologicals	NB410-28088
Armenian Hamster IgG	Mouse/Human	*a*	Biolegend	400902

a. IgG isotype controls were diluted to the same concentration of primary antibody for each biomarker

Biotinylated secondary	Species	Dilution	Vendor	Catalog #
Goat Anti-Rat IgG, Biotin-SP	Mouse/Human	1:750	Millipore Sigma	AP183B
Goat Anti-Rabbit IgG, Biotin-SP	Mouse/Human	1:500	Millipore Sigma	AP132B
Goat Anti-Mouse IgG, Biotin-SP	Mouse/Human	1:500	Millipore Sigma	AP124B
Goat Anti-Armenian hamster IgG, Biotin	Mouse/Human	1:250	Biolegend	405501
Rabbit Anti-Goat IgG, Biotin	Mouse/Human	1:750	ThermoFisher	A16146

Supplementary Table 6. Antibodies used for single EV imaging.

Primary antibody	Dilution	Vendor	Catalog #	Isotype
CD9	1:50	Biolegend	124802	Rat IgG2a
CD63	1:50	Biolegend	143902	Rat IgG2a
PAX8	1:50	ProteinTech	60145-4-Ig	Mouse IgG
CA125	1:100	LsBio	LS-C408274	Rabbit IgG polyclonal
VCAN	1:100	Bioss	BS-2533R	Rabbit IgG polyclonal
TNC	1:100	LsBio	LS-C413317	Rabbit IgG polyclonal
FOLR1	1:100	LsBio	LS-C119975	Rabbit IgG polyclonal
HE4	1:100	LsBio	LS-C409035	Rabbit IgG polyclonal
**Secondary antibody**				
Anti-Rat AlexaFluor647	1:400	Abcam	ab150155	-
Anti-Mouse AlexaFluor488	1:400	Abcam	ab150105	-
Anti-Rabbit AlexaFluor594	1:400	Abcam	ab150076	-
